# Spin-Forbidden Carbon–Carbon Bond Formation in Vibrationally Excited α-CO

**DOI:** 10.1021/acs.jpca.2c01168

**Published:** 2022-04-05

**Authors:** Jessalyn A. DeVine, Arnab Choudhury, Jascha A. Lau, Dirk Schwarzer, Alec M. Wodtke

**Affiliations:** † Abteilung für Dynamik an Oberflächen, Max-Planck-Institut für Multidisziplinäre Naturwissenschaften, Am Faßberg 11, 37077 Göttingen, Germany; ‡ Institute for Physical Chemistry, Georg-August Universität Göttingen, Tammannstaße 6, 37077 Göttingen, Germany

## Abstract

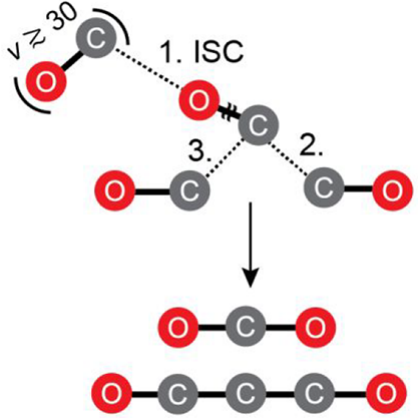

Fourier transform infrared spectroscopy of laser-irradiated cryogenic crystals shows that vibrational excitation of CO leads to the production of equal amounts of CO_2_ and C_3_O_2_. The reaction mechanism is explored using electronic structure calculations, demonstrating that the lowest-energy pathway involves a spin-forbidden reaction of (CO)_2_ yielding C(^3^P) + CO_2_. C(^3^P) then undergoes barrierless recombination with two other CO molecules forming C_3_O_2_. Calculated intersystem crossing rates support the spin-forbidden mechanism, showing subpicosecond spin-flipping time scales for a (CO)_2_ geometry that is energetically consistent with states accessed through vibrational energy pooling. This spin-flip occurs with an estimated ∼4% efficiency; on the singlet surface, (CO)_2_ reconverts back to CO monomers, releasing heat which induces CO desorption. The discovery that vibrational excitation of condensed-phase CO leads to spin-forbidden C–C bond formation may be important to the development of accurate models of interstellar chemistry.

## 1 Introduction

Spectroscopic identification of extraterrestrial carbon-containing polyatomic molecules has long sparked the imagination of astrochemists, who suggest that the origins of terrestrial life may involve extraterrestrial chemistry.^1−6^ As the most abundant carbon-containing compound in the universe,^7,8^ reactions of carbon monoxide that lead to carbon–carbon bond formation play a key role. Astronomical CO is found in the gas phase, but is also present as ices in the cold interstellar clouds postulated to be the birthplace of the “molecules of life”.^9^ To unravel the chemical mechanisms taking place in these pockets of the universe, experimentalists have induced reactions in laboratory analogs of astronomical CO ices by bombardment with highly energetic (∼10–100 keV) photons, electrons, and ions, mimicking the effects of background radiation in the interstellar medium.^10−16^ Under these harsh conditions a wide array of carbon-rich molecules are formed, including C_*n*_, C_*n*_O, and C_*n*_O_2_ with up to 7 carbon atoms. One of the simplest stable products containing carbon–carbon bonds is carbon suboxide, C_3_O_2_ (O=C=C=C=O), which has been proposed to be present in Comet Halley.^17^ Like CO, C_3_O_2_ is a fundamentally stable, closed-shell molecule; unlike CO, it is much more reactive, and could provide a carbon source for producing more complex organic molecules.^18,19^

The most thorough account of chemical transformations in irradiated CO ices is the mechanistic framework developed by Jamieson and co-workers.^14^ They proposed C_3_O_2_ is formed from a highly excited (yet ill-defined) CO* molecule, producing reactive carbon atoms which go on to recombine with CO through reactions [1–3].
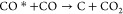
1
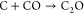
2
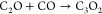
3However, the harsh conditions of those experiments mean that a number of other processes are also possible. This chemical complexity makes it difficult, if not impossible, to establish the fundamental mechanism of C–C bond formation in CO ices.

Vibrational energy pooling (VEP) offers an attractive alternative for depositing energy into condensed-phase CO.^20,21^ Laser-excitation of CO(*X̃* ^1^Σ^+^,*v* = 0 → 1) induces near-resonant vibrational energy transfer between neighboring molecules in quantum states *v* and *w*,
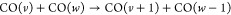
This process has been studied in detail with infrared laser-induced fluorescence (IR-LIF).^22,23^ Through a base-camp mechanism occurring over a few microseconds, CO molecules initially in the *v* = 1 state pool their energy, producing a dilute population of highly excited molecules in levels with *v* ≤ 37 (corresponding to energies of up to ∼7 eV). Curiously, the rate of infrared emission drops discontinuously starting at *v* ≈ 30, indicating an energy threshold above which CO vibrational excitation experiences another fate.

In this work, we use Fourier transform infrared (FTIR) absorption spectroscopy of laser-irradiated α-CO crystals to show that highly vibrationally excited CO produced by VEP reacts to form CO_2_ and C_3_O_2_ in equal amounts. Possible reaction pathways for the lowest-lying singlet and triplet states are explored using electronic structure theory, modeling the relevant reactive species in the crystal as a gas-phase CO-dimer. These calculations show that formation of the observed products likely relies on transitions to the triplet surface via intersystem crossing (ISC) of a CO dimer structure, energetically accessible by the highest-*v* states known to be populated through VEP. Trajectories remaining on the singlet pathway are found to reconvert to 2CO from the critical dimer structure in an exothermic process, releasing heat that drives desorption of CO. Together, the experimental and theoretical results provided here explain the energy thresholds observed in VEP, and shine new light on the mechanisms by which carbon–carbon bonds can form in condensed phase CO.

## 2 Methods

### 2.1 Experimental Section

The apparatus and procedure used to prepare and characterize thin, well-ordered samples of α-CO on NaCl in ultrahigh vacuum (UHV) conditions has been described in previous work.^24,25^ In these experiments, a UHV-cleaved NaCl(100) surface is thermally coupled to a dual-stage helium cryo-cooler and positioned within a liquid nitrogen cooled thermal shield. A cryogenic temperature controller is connected to the sample holder, allowing for well-determined surface temperatures as low as 7 K. Samples are prepared by directing a fixed number of molecular beam pulses of CO gas onto the NaCl crystal at a temperature of 22 K. After dosing, the sample is slowly cooled to 7 K, forming a well-ordered crystal structure. Initial sample characterization is then performed using an FTIR spectrometer operating in external mode with a liquid nitrogen cooled InSb detector. The FTIR beam impinges upon the sample at an incidence angle of 45°, and a wire grid polarizer allows for measurement of *s*- and *p*-polarized spectra, using a background spectrum corresponding to the NaCl(100) crystal at a temperature of 22K.

Three samples prepared with initial coverages of ∼150–500 layers of CO were used to obtain the results presented in this work. To minimize impurities which may contribute to the chemical activity of the samples, the CO dosing gas (either isotopically pure ^12^C^16^O or natural abundance) was precooled using a pentane ice bath held at appox. −130 °C. The initial sample characterization, yielding *s*- and *p*-polarized spectra consistent with previous measurements,^26^ confirmed the formation of thin well-ordered α-CO samples with minimal impurities.

After sample preparation and characterization, an excite-probe cycle was performed to monitor the changes that occur following laser excitation. Laser excitation was achieved using the difference frequency mixing (DFM) setup described previously.^22,23,27^ Here, a tunable dye laser is pumped by the second harmonic (532 nm) of a seeded 10 Hz Nd:YAG laser; tunable infrared light is then generated by mixing the dye laser output (∼850–890 nm) with the Nd:YAG fundamental (1064 nm) in a LiIO_3_ crystal. This crystal is held at a temperature of 100 °C to minimize absorption of atmospheric water. An electronic shutter situated at the output of the DFM setup and coupled to the FTIR data acquisition software was used to expose the sample to a fixed number of laser pulses, after which a *p*-polarized FTIR spectrum was taken. Due to the limited detection range of the InSb detector, absorption spectra are reported for photon energies above 1900 cm^–1^. The excite-probe procedure was repeated until the sample had been exposed to 60 000 laser shots, after which a final *s*-polarized spectrum was acquired. As shown in Figure S1 of the Supporting Information (SI), the only spectral features considered in this work which show polarization dependence are the CO optical modes.

For the measurements reported here, the DFM setup was tuned to excite the transverse optical (TO) mode of α-^12^C^16^O (2138.6 cm^–1^) with typical pulse energies of ∼200 μJ (∼10 ns pulse width). The excitation beam was focused onto the sample with a spot size of diameter 0.5 mm, resulting in fluence that is sufficient to saturate the *v* = 0 → 1 transition of CO and initiate the VEP process.^23^ To match the spatial extent of the excited region with the area probed by the FTIR beam (2 mm diameter), the pump laser spot was scanned periodically over the sample using a piezo-controlled mirror, leading to irradiation of a ∼ 3.1 × 2.2 mm^2^ area of the sample.

The measurements reported here took several hours to complete, over which time the InSb detector exhibited some baseline drift. The spectra were baseline-corrected by identifying regions in which there are no spectral features, and obtaining an interpolated background curve that was then subtracted (see Figure S2 in the SI).

### 2.2 Computational

Electronic structure calculations were carried out using QChem version 5.4.^28^ First, density functional theory (DFT) was used to obtain geometries and harmonic frequencies for the lowest singlet and triplet states of the reactants and products of reactions [1–3] at the ωB97M-V/6-311+G* level.^29^ This functional—a combinatorically optimized, range-separated hybrid, meta-GGA density functional with VV10 nonlocal correlation—was chosen due to its relatively high position on the “Jacob’s ladder” of DFT,^30^ along with its relatively low computational expense and compatibility with the various computational methods used here. Reactant and product geometries were connected using freezing string method (FSM) calculations within the ωB97M-V/6-311+G* framework,^31^ and a transition state (TS) optimization on the highest-energy FSM point was then performed for all six processes to determine which elementary steps are barrierless. Single-point calculations on the optimized reactant, TS, and product geometries were then performed at the CCSD(T)/def2-qZVP level of theory to obtain more reliable energies. To ensure the validity of this single-reference approach, the *T*_1_ diagnostic was used, which considers the ratio of the norm of the T_1_ CCSD amplitudes to the number of electrons;^32^ these values are given in Table S1 of the SI along with the absolute energies for all species considered here. Optimized geometries, harmonic frequencies, and reaction energies are provided in Tables S2–S4.

For closer inspection of the saddle point geometries, intrinsic reaction coordinate^33^ and spin-flip time-dependent DFT calculations^34^ were carried out using the chosen ωB97M-V/6-311+G* framework; this functional and basis set was also used to calculate the minimum energy crossing point (MECP) between the singlet and triplet surfaces of [1].^35^

For calculation of intersystem crossing rates, spin–orbit coupling (SOC) matrix elements were computed using the equation-of-motion coupled-cluster (EOM-CCSD) method with the 6-311G basis set.^36,37^ The values used in rate calculations correspond to the total one-electron matrix elements of a Breit-Pauli Hamiltonian, obtained directly from the QChem output. Derivative coupling elements were obtained by performing these calculations on geometries displaced from equilibrium along the normal modes given by the ωB97M-V/6-311+G* frequency analysis, and numerically differentiating the resultant SOC curves.

## 3 Results

### 3.1 Experimental Section

**Figure 1:**
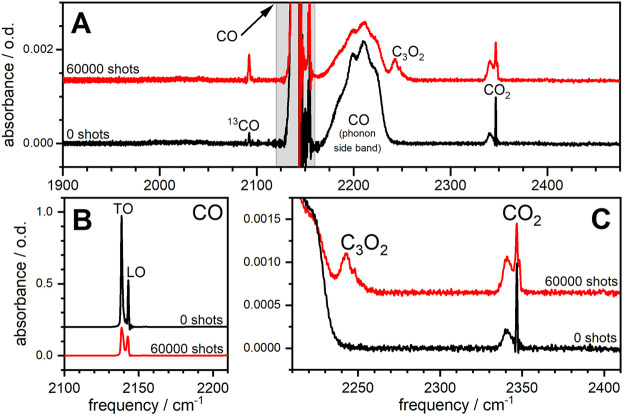
Initial and final FTIR spectra of vibrationally excited α-CO. (A) Optical density (o.d.) of a ∼300-layer sample held at 7 K before (black) and after (red) excitation with 60 000 laser pulses. (B) CO absorption features. (C) CO_2_ and C_3_O_2_ absorption features. The large peak at the low-frequency end of this panel corresponds to the CO phonon sideband in panel A.

Figure 1A shows FTIR spectra for a ∼300-layer sample of α-CO before and after laser irradiation; similar results are obtained for samples with varying initial CO layer thickness (see Figure S3 of the SI). Prior to laser excitation, FTIR spectra are dominated by the ^12^C^16^O transverse and longitudinal optical (TO and LO) modes at ∼2140 cm^–1^ and a CO phonon sideband at ∼2200 cm^–1^.^26^ Weaker features in the pre-excitation spectra are attributed to minor impurities (^13^CO, CO_2_) in the dosing gas.

Excitation of the α-CO TO mode (2138.6 cm^–1^) with 60 000 laser pulses induces three primary changes in the spectra. First, the CO absorption in the ∼2100–2200 cm^–1^ region is considerably depleted (Figure 1B). Second, the CO_2_ antisymmetric stretching band intensity increases (Figure 1C). Finally, we see the appearance of a new feature at ∼2250 cm^–1^, which arises from carbon suboxide, as has been discussed in previous reports (Section S1, Figures S4 and S5 of the SI).^11^ We see no evidence for production of any other new species beyond CO_2_ and C_3_O_2_.

**Figure 2:**
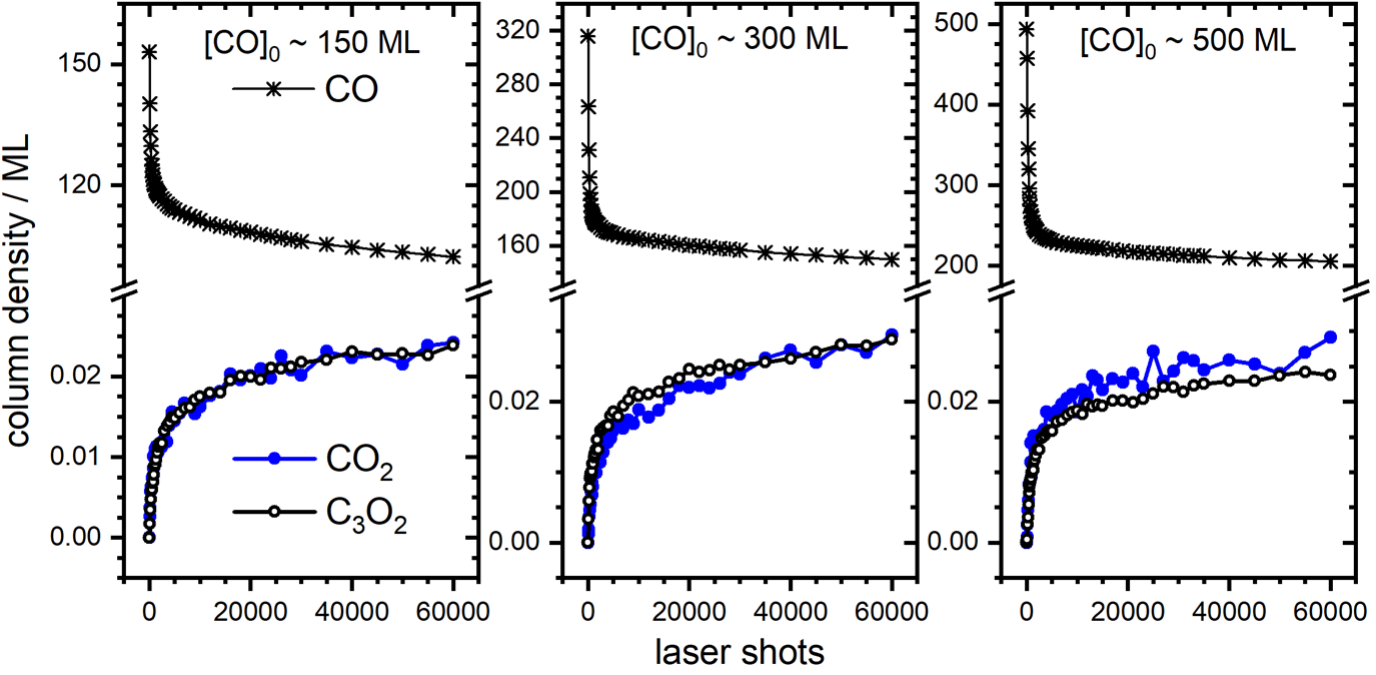
Evolution of CO, CO_2_, and C_3_O_2_ for samples with varying initial thickness. Column densities are reported in units of monolayer (ML) using the 2D density associated with a single layer of pure α-CO (6.27 × 10^14^ molecules cm^–2^).^38^ The CO_2_ results have been corrected for the initial CO_2_ impurity in the unexcited sample, as well as the amount that adsorbs over the course of the experiment due to residual gas in the UHV chamber (see Figure S6).

From these spectra and data presented in Table S5, we obtain column densities for CO, CO_2_, and C_3_O_2_ as a function of the number of laser pulses used in the excite-probe experiment (Section S2a of the SI); see Figure 2. The growth curves of CO_2_ and C_3_O_2_ mirror one another—in other words, for every CO_2_ molecule produced, a single C_3_O_2_ molecule is formed. This is consistent with reactions [1–3] as the elementary steps needed for product formation, so that the overall reaction is given by
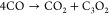


We now note two important observations that we will revisit later. First, the extent of CO depletion is far greater than can be explained solely by conversion to the photoproducts, which accounts for <0.2% of the total loss of CO in all three samples (Section S2b, Table S6, Figure S7). This indicates that the majority of CO is lost to desorption, consistent with observed spikes in the UHV chamber pressure during laser excitation. We also note a saturation effect; for both reactants and products, the extent of laser-induced change is initially rapid, slowing dramatically after about 5000 laser shots.

### 3.2 Reaction Pathway Calculations

To better understand the results in Figures 1 and 2, electronic structure calculations were carried out for the overall mechanism given by [1–3]. Given the low density of high-energy CO molecules produced by VEP, the CO molecules added at each step are assumed to be in the ground *X̃* ^1^Σ^+^ state, and we do not consider production of the triplet state of CO_2_ as relevant given the exceedingly high calculated excitation energy of this state (∼4.5 eV). We also do not consider CO_2_ formation in a two-step process initiated by direct dissociation of CO,
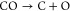

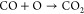
as a contributor to our mechanism. This was taken to be an insignificant pathway in the high-energy experiments on CO ices^14^ due to the absence of products with more than 2 oxygen atoms; given the lower energies used in the current work, it is unlikely that the dissociation limit of CO(*X̃* ^1^Σ^+^)—ca. 10 eV^39^—will be reached in our system.

**Figure 3:**
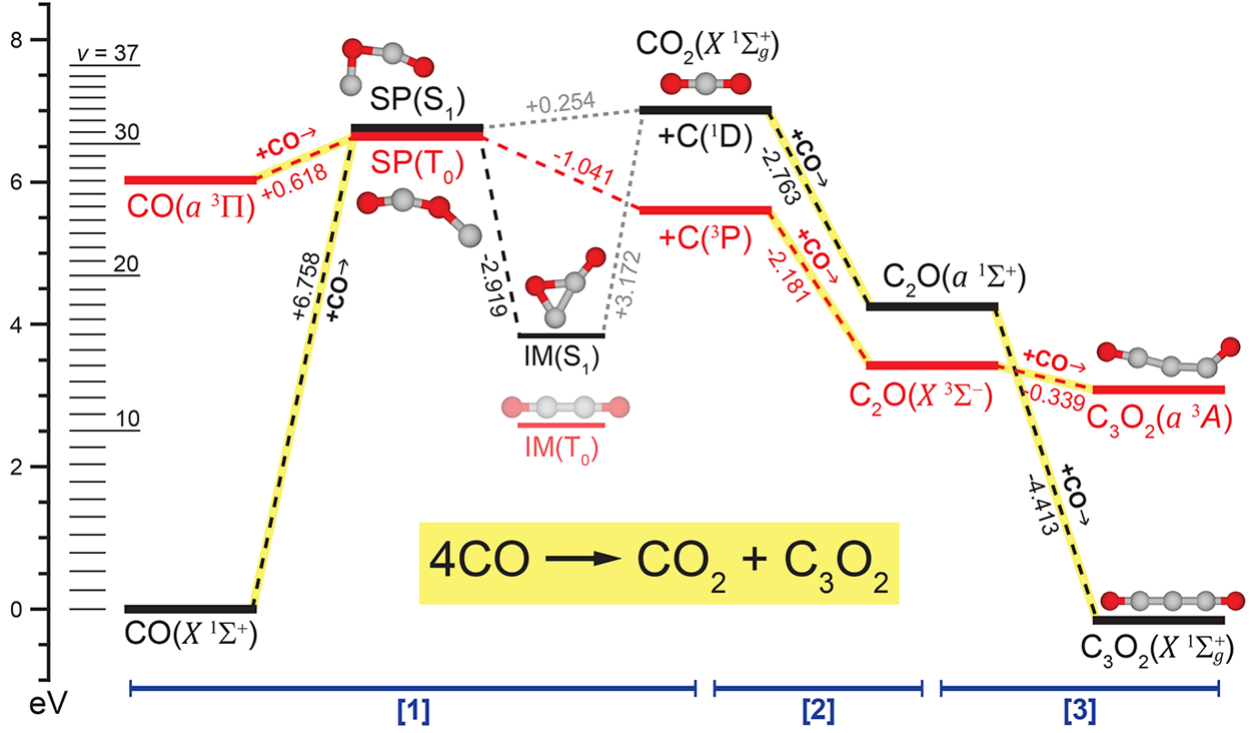
Reaction mechanism [1–3] describing formation of CO_2_ and C_3_O_2_ from vibrationally excited CO. Singlet and triplet pathways are shown in black and red, respectively, and energy differences between states connected by dashed lines are given in units of eV. Energies for each step in the overall reaction are obtained from CCSD(T)/def2-qZVP results with ωB97M-V/6-311+G* zero-point correction. Steps where CO is consumed are highlighted in yellow. For reference, energies of CO(*X̃* ^1^Σ^+^,*v*) + 3CO(*X̃* ^1^Σ^+^, 0) are shown for *v*-levels populated through VEP.^22^

With this in mind, our computational endeavors yield the energy diagram of Figure 3. With the exception of the reactants of [1] and the products of [3], the triplet pathway is always lower in energy, implying the existence of at least two crossing points. For both singlet and triplet [1], the TS optimizations led to true saddle points (SPs) whose geometries agree with those reported previously;^40^ the triplet saddle point SP(T_0_) is ∼0.1 eV lower in energy than the corresponding singlet state, indicating that the first singlet/triplet crossing occurs prior to the TS to [1]. Reactions [2 and 3] were found to be barrierless for both spin states, with the TS optimizations leading to either the reactant or product geometries. The lack of barriers for [2–3] is consistent with the absence of experimentally observed C_2_O spectral features, as well as the similar yields of CO_2_ and C_3_O_2_ (Figure 2); in this energetic picture, as soon as CO_2_ is formed, the system proceeds without a barrier to C_3_O_2_, regardless of spin. We also note in passing that reaction [3] has been found to be barrierless in a CO matrix,^41^ consistent with our findings.

Although SP(S_1_) does correspond to a saddle point in the DFT treatment used here insofar as it possesses a single imaginary frequency, there are several aspects of this geometry that arouse suspicion of its relevance to reaction [1]. While the structures of SP(T_0_) and SP(S_1_) are similar to those obtained in an MP2 treatment reported previously,^40^ this earlier work placed SP(S_1_) at a higher energy than the CO_2_(*X̃* ^1^Σ_*g*_^+^) + C(^1^D) products. In contrast, we find that the conversion of SP(S_1_) to the singlet products of [1] has a positive barrier of ca. 0.25 eV (Table S4). Given this discrepancy, repeated calculations were performed at various levels of theory, as discussed in Section S3 (Figure S8) of the SI. We find that the energetic picture given by Figure 3 is fairly robust, and proceed with the assumption that the SP(S_1_) geometry indeed lies below the products to reaction [1] on the singlet pathway.

**Figure 4:**
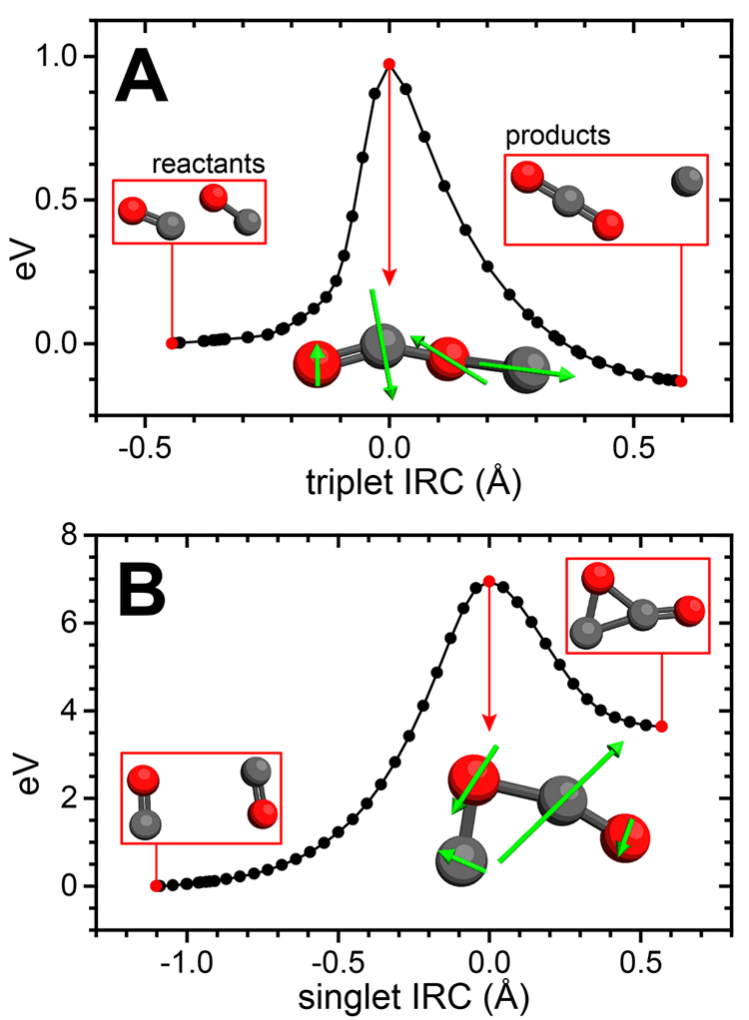
IRC calculations for (A) SP(T_0_) and (B) SP(S_1_). Energies relative to the “reactant” geometries are plotted versus root-mean-square displacement from the SP geometries. The SP structures are shown with green arrows depicting the atomic displacements associated with their imaginary frequencies.

To assess the relevance of a saddle point structure to a particular reaction, one may perform an intrinsic reaction coordinate (IRC) calculation on the transition state geometry to determine the “reactants” and “products” implied by the imaginary frequency obtained in a normal-mode analysis.^33^ Performing such an IRC calculation on SP(T_0_) (Figure 4A), using our chosen ωB97M-V/6-311+G* framework, shows the expected behavior for a transition state of [1], leading to formation of 2CO and CO_2_ + C in the reverse and forward directions, respectively. In contrast, the IRC calculation for the singlet SP (Figure 4B) leads to a cyclic intermediate, IM(S_1_), rather than the CO_2_ + C products for [1]; in the triplet state, this structure converges to a linear OCCO geometry lower in energy than IM(S_1_) (Figure 3). Thus, the identified saddle point SP(S_1_) does not correspond to a true TS to reaction [1], in contrast to SP(T_0_).

The possibility of accessing CO_2_ + C_3_O_2_ via the IM(S_1_) intermediate was also explored (see Section S4a of the SI)—these calculations yield no energetically viable pathway for formation of the observed products, while reconversion to 2CO molecules is barrierless (Figures S9 and S10). This tendency toward dissociation is further illustrated by the molecular dynamics simulations described in Section S4b of the SI, which show that even when generated with considerable initial energy, IM(S_1_) rapidly dissociates into 2CO fragments (Figures S11–S14) rather than accessing a higher-energy channel.

### 3.3 Singlet/Triplet Crossing for a CO Dimer

The computational results detailed in the previous section show that the experimentally observed products are not readily formed on the singlet potential energy surface (PES). One might expect that traversal of SP(S_1_), which resembles a previously reported TS to [1],^40^ would provide a spin-allowed pathway to products. However, as noted above, SP(S_1_) lies ∼0.25 eV below the CO_2_ + C(^1^D) bottleneck for C_3_O_2_ formation on the singlet surface. Indeed, while the IRC calculation for SP(T_0_) yields the expected behavior for a TS to [1] (Figure 4A), we find that SP(S_1_) correlates with a cyclic C_2_O_2_ intermediate IM(S_1_) (Figure 4B), which lies ∼3 eV below the expected CO_2_ + C(^1^D) products and can readily convert back to the 2CO reactant well (Figure S9, Section S4b). These results strongly suggest that the CO_2_ and C_3_O_2_ products observed in this work are formed following a spin-flip to the triplet PES and that spin-allowed chemical processes on the singlet PES are not productive, leading back to reactants plus heat.

**Figure 5:**
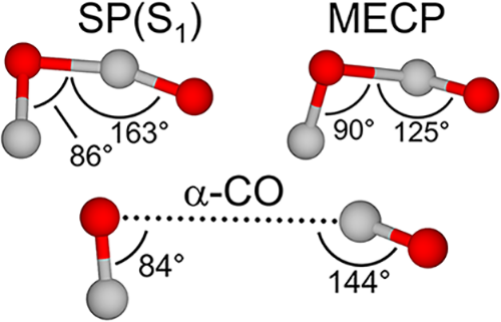
CO dimer geometries. The SP(S_1_) and MECP geometries obtained at the ωB97M-V/6-311+G* level of theory (top) show a resemblance to the geometry of a dimer within the α-CO crystal lattice (bottom).^42^ A more detailed comparison of these geometries may be found in Figure S16 and Table S7 of the SI.

With this in mind, we identified the MECP of the singlet and triplet surfaces, which lies ∼1 eV below SP(T_0_) (Section S5, Figure S15) and strongly resembles the SP(S_1_) geometry. Interestingly, the energy of the MECP at the CCSD(T)/aug-cc-pVTZ level of theory places this singlet geometry only 0.1 eV above the triplet CO_2_ + C(^3^P) products to [1]. Comparison of the MECP geometry to a dimer within the α-CO lattice^42^ also shows a striking orientational similarity, as shown in Figure 5. Only a translation of one CO molecule toward the other is needed to convert the structure of the dimer within the crystal to that of the MECP; we postulate that this is possible for high-*v* vibrationally excited CO, and may also be facilitated by phonon excitations in the surrounding crystal lattice. This argues that the structural constraints of the α-CO lattice do not restrict access to the geometry of the singlet/triplet MECP, and preorientation within the crystal may even promote ISC.

### 3.4 Intersystem Crossing Rates

To test the hypothesis that products are formed by a spin-forbidden reaction mechanism, we calculated ISC rates for a CO dimer. We use the time-independent formulation of Fermi’s Golden Rule to calculate the rate of transitions between vibronic levels belonging to electronic manifolds of differing spin, as detailed in Section S6a of the SI. While the SOC perturbation which enables transitions between these states is typically treated as a purely electronic operator,^43^ the observation that the singlet and triplet surfaces in Figure 3 cross motivates a spin-vibronic (SV) mechanism for ISC, so that we include nuclear effects on the calculated SOC elements.^44^ We consider that the role of the surrounding CO crystal is to ensure energy conservation, so that the energy discrepancy between initial and final states is accounted for by the CO phonon bath (Section S6b of the SI).

We again note the structural similarities between SP(S_1_) and the MECP (Figure 5), so that SP(S_1_) may represent a point at which the triplet surface can be accessed. We thus employ a reduced-dimensionality—i.e., neglecting modes with imaginary frequencies—SV-ISC mechanism to consider transitions between vibrational states of the two SP geometries,
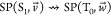
as detailed in Section S7a (Figures S17–S18, Table S8) of the SI. Resultant nonzero rates are provided in Tables S9 and S10. These calculations show that within ∼500 fs, the vibrational ground state of SP(S_1_) can undergo a spin-flip transition; we consider this to be representative of the time scale on which ISC can occur in dimer structures accessed in the crystal (see Section S7b). Comparing this to the ∼20 fs lifetime of SP(S_1_) implied by its imaginary frequency, we estimate that ∼4% of CO-pairs that access SP(S_1_) may undergo ISC into the triplet manifold.

## 4 Discussion

With these results, we suggest a simple picture for the observed chemical evolution of vibrationally excited CO ices. Laser-excitation of a fresh sample induces VEP, producing a sparse population of highly vibrationally excited CO(*X̃* ^1^Σ^+^,*v*). This provides access to high-energy regions of the singlet PES for reaction [1], so that a molecule at the upper-end of the *v*-states populated through VEP can unite with a neighboring CO molecule and form a dimer resembling the MECP or SP(S_1_) structures identified in our gas-phase model. This dimer is unlikely to proceed to form the observed CO_2_ + C_3_O_2_ products on the singlet surface. However, it may undergo rapid ISC and access the triplet pathway, possibly via a geometry resembling the SP(T_0_) structure that can proceed to form CO_2_ + C(^3^P) (Figure 4A). The triplet dimer may instead dissociate to form 2 CO fragments; this could still result in formation of our observed products, given gas-phase experiments showing that CO(*ã* ^3^Π) reacts to produce CO_2_ + C_3_O_2_ following a sequence similar to [1–3].^45−48^ Once generated through [1], C(^3^P) proceeds without a barrier to react with another CO, forming C_2_O(*X̃* ^3^Σ^–^), which subsequently reacts with CO yielding C_3_O_2_. Thus, the formation of carbon–carbon bonds in our samples is facilitated by ISC of a CO dimer, produced from the energetic reaction centers generated through VEP.

Of course, spin–orbit coupling in light molecules like CO is typically small. So, the reader should question why a reaction on the singlet surface is unimportant, especially given the estimation that in the gas-phase model, only ∼4% of CO pairs accessing SP(S_1_) undergo a transition to the triplet surface. The insight needed to address this question comes from our IRC calculations showing that the imaginary frequency of SP(S_1_) correlates with formation of IM(S_1_) (Figure 4B), which must then surpass a ∼3 eV barrier to form CO_2_ + C(^1^D) (Figure S9). We consider this reaction impossible at the low temperatures of our experiments; indeed, constant-energy molecular dynamics trajectories of IM(S_1_) initialized with the kinetic energies associated with its direct formation from SP(S_1_) show that even without a cold environment rapidly removing heat from this molecule, it dissociates to 2 CO fragments (Section S4b). Thus, in the case that a dimer resembling SP(S_1_) does not undergo ISC into the triplet manifold, it will ultimately reconvert to the reactant well in an exothermic process. The heat released by this dissociation likely drives the desorption observed to dominate the depletion of CO in our samples (Figure 2).

Our gas-phase calculations indicate that a CO dimer geometry—possibly resembling the MECP or SP(S_1_)—is a critical point for the observed changes in our samples, as it serves as the point of access for product formation (via ISC to the triplet surface) as well as CO desorption (via conversion to the cyclic IM and subsequent dissociation). To produce CO with the levels of vibrational excitation necessary to access this structure, VEP occurs via a base-camp mechanism between molecules many lattice sites away; previous work has shown that consideration of interactions between molecules up to 8 lattice sites away is required to account for population of *v* = 22.^23^ To produce populations in the states relevant to this work (*v* ≥ 31), we speculate that even longer-range intermolecular interactions are essential. Hence, fractional polyatomic impurities on the 10^–3^ level are likely to interrupt the VEP process by acting as vibrational energy sinks. Deposition of vibrational energy into such polyatomics will be followed by rapid intramolecular vibrational redistribution, reallocating the energy along low-frequency modes that easily couple to the phonon bath of the surrounding crystal. Therefore, it is not an accident that the product formation saturates when they constitute <0.1% of the total sample (Figure S7); as the photoproducts accumulate in the CO matrix, the efficiency of VEP is diminished such that fewer CO pairs access the critical dimer geometry, resulting in the observed saturation behavior. The mechanistic picture provided here is thus consistent with both of the finer points highlighted in Section 3a regarding the kinetic traces in Figure 2.

The importance of dimer geometries, such as the gas-phase MECP and SP(S_1_), in the dynamics of vibrationally excited CO is further supported by considering previous results of IR-LIF experiments probing VEP in the α-CO system. Figure 3 shows the energies of CO vibrational levels that have been observed in such experiments (*v* ≤ 37);^22^ we note that the energy of our calculated SP(S_1_) structure lies between *v* = 31 and *v* = 32, which is remarkably close to the *v*-levels for which infrared fluorescence begins to become efficiently quenched (see Figure 7 of ref (22)). With the ISC rates calculated for transitions out of the SP(S_1_) manifold, we confirm prior suspicion that this quenching of emission from higher *v*-states is connected to vibration-to-electronic energy transfer (VEET) into an excited triplet state. However, only a small fraction of the SP(S_1_) dimers will undergo ISC, and thus the observed quenching of higher-*v* states in LIF experiments arises from a more nuanced picture than a simple electronic transition—namely, in addition to enabling VEET, SP(S_1_) can provide access to the metastable IM(S_1_) which effectively converts CO vibrational energy to heat.

Finally, we emphasize the somewhat surprising result that infrared-excitation of condensed-phase CO leads to production of molecules that are formed by high-energy irradiation of CO ices. These high-energy experiments involve continuous exposure to energetic particles resulting in a high concentration of reaction centers in a range of vibronic states, the identities of which are not always readily assumed. Because of the nonspecificity of such radiation, products formed at these centers can be subsequently excited, further complicating the dynamics and leading to production of a wide range of carbon-rich species. In the approach used here, the only possible excitation is CO(*X̃* ^1^Σ^+^,*v* = 0 → 1), so that reaction centers are produced indirectly (through VEP) and in low concentrations.^23^ As such, product molecules generated by reactions [1 and 2] are unlikely to be formed in close proximity, limiting secondary reactions. Further, the lower energies allow for more confident assignment of the states involved in chemical transformations; thus, the milder conditions used here enable a clear understanding of the physical processes underlying [1–3].

## 5 Conclusions

Vibrational excitation of thin α-CO crystals at 7 K is found to induce carbon–carbon bond formation, and the pathways leading to this chemistry are explored using electronic structure theory. Together, the experimental and theoretical work presented in this paper strongly support a mechanism wherein highly vibrationally excited CO molecules produced by VEP access a critical dimer geometry that effectively removes vibrational energy from the VEP process. Chemical transformations are unlocked by ISC in this dimer, forming CO_2_ + C(^3^P) by a spin forbidden reaction [1] between two CO molecules. C(^3^P) subsequently recombines with CO in two barrierless steps, [2–3], to produce C_3_O_2_. In the event that the triplet pathway is not accessed, the dimer ultimately reconverts back to 2CO molecules, releasing heat that drives desorption of CO. These processes are reflected in the chemical evolution of laser-irradiated CO samples monitored using FTIR, which shows that CO depletion is primarily driven by desorption, and equal amounts of CO_2_ and C_3_O_2_ are formed.

While the molecules observed here are known to be formed in similar CO crystals irradiated with high-energy particles, we show that the dynamics of vibrationally excited condensed-phase CO is an alternative means by which reactive centers may be produced, leading to chemistry resembling that occurring in astronomical CO ices. Due to the properties of the base-camp mechanism of VEP,^23^ reaction centers are dilute and secondary reactions are suppressed; as a result, a simpler set of observables is obtained, allowing a clear understanding to emerge. Ultimately, we show that vibrationally excited CO is capable of carbon–carbon bond formation via a spin-forbidden reaction producing CO_2_ and C_3_O_2_.

## Supporting Information Available

Full FTIR spectra for all three samples studied; polarization-dependence of the CO, CO_2_, and C_3_O_2_ spectral features; tabulated results of reaction pathway calculations including absolute energies, geometries, and vibrational frequencies; discussion of spectral assignments; quantitative analysis of FTIR spectra, including analysis of product yields comparison of reaction pathway calculations for different levels of theory; further calculations regarding IM(S_1_) and SP(S_1_), including stationary state calculations and molecular dynamics simulations; details regarding the MECP calculation; model used for calculating ISC rates; and application of this model to the CO dimer structure represented by SP(S_1_) (PDF)
