# Supplementary material for: Spin-Forbidden Carbon–Carbon Bond Formation in Vibrationally Excited α-CO
Source: J Phys Chem A. 2022 Apr 5;126(14):2270–7. doi: 10.1021/acs.jpca.2c01168 (PMC9014413; doi:10.1021/acs.jpca.2c01168)
Supplement: Supplementary file 1 — jp2c01168_si_001.pdf [file jp2c01168_si_001.pdf]

Supporting Information for “Spin-Forbidden Carbon-Carbon Bond Formation in Vibrationally-Excited α-CO”

Jessalyn A. DeVine,[†] *Arnab Choudhury,*[†,‡] *Jascha A. Lau,*[†,‡,#] *Dirk Schwarzer,*[†] *Alec M. Wodtke.*[†,‡*]

[†] Abteilung für Dynamik an Oberflächen, Max-Planck-Institut für Multidisziplinäre Naturwissenschaften, Am Faßberg 11, 37077 Göttingen, Germany.

[‡] Institute for Physical Chemistry, Georg-August Universität Göttingen, Tammannstr. 6, 37077 Göttingen, Germany.

*Correspondence to: alec.wodtke@mpibpc.mpg.de

Contents

S1. Spectral Assignments …… S5
S2. Quantitative Analysis of FTIR Spectra
  a. Calculation of Column Densities …… S7
  b. Yield Analysis of Concentration Data …… S8
S3. Comparison of Different Levels of Theory …… S11
S4. Further Calculations Regarding SP(S_1) and IM(S_1)
  a. Stationary State Calculations…… S13
  b. Molecular Dynamics Simulations…… S14
S5. Curve-Crossing in Reaction [1] …… S17
S6. Intersystem Crossing Rates
  a. Fermi’s Golden Rule Model…… S19
  b. Treatment of the Phonon Density of States…… S20
S7. ISC in a CO Dimer
  a. SP(S_1) ⇝ SP(T_0) …… S21
  b. Other CO Dimer Structures…… S25
References……S25

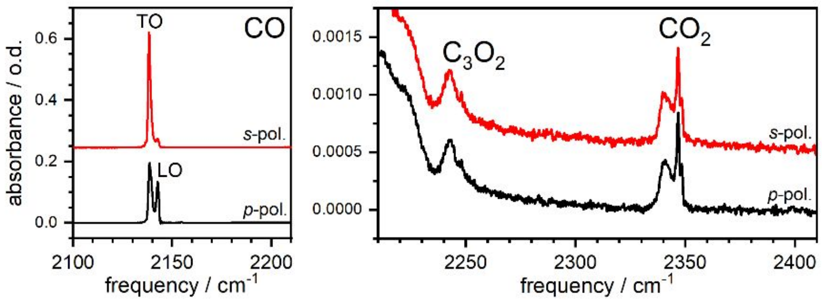

Figure S1. Polarization-dependent FTIR spectra. Absorption features assigned to (left) CO and (right) C_3O_2/CO_2 are shown. Red and black traces correspond to *s*- and *p*-polarized spectra, respectively. Data correspond to the ca. 300 layer sample represented in Fig. 1 but do not include the background subtraction illustrated by Fig. S2.

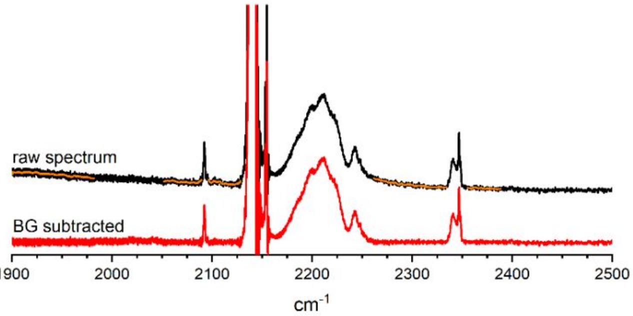

Figure S2. Illustration of background subtraction of FTIR spectra. The black trace is an uncorrected *p*-polarized FTIR spectrum of a ~300 layer $^{12}C^{16}O$ sample acquired after the sample had been exposed to 60000 laser shots. Orange lines show the portions of the spectrum that were chosen to form the background interpolation, resulting in the subtracted spectrum shown in red.

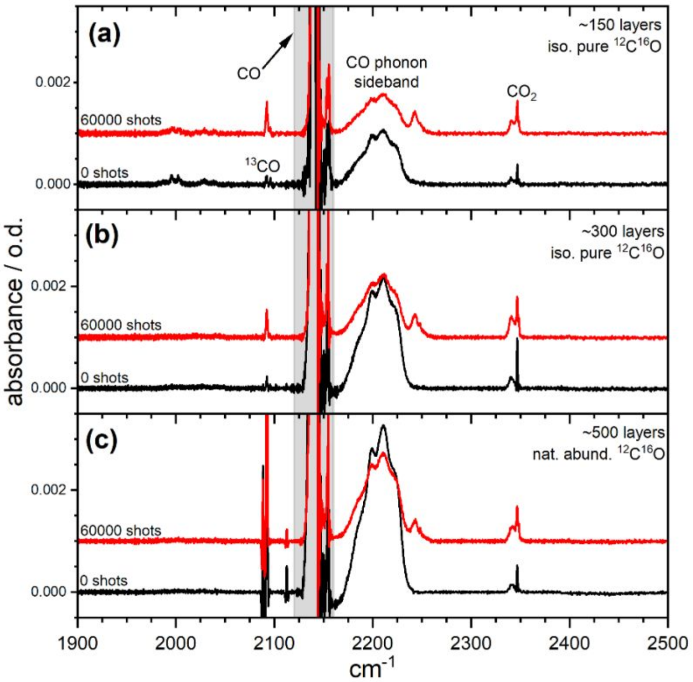

Figure S3. Initial and final *p*-polarized FTIR spectra for three samples of α-CO subjected to the excite-probe procedure. The initial CO coverage and isotopic purity of the dosing gas are indicated in the top-right corner of each panel. The weak features at ca. 2000 cm^{-1} in (a) are due to iron carbonyl impurities that were not fully removed from the dosing gas by the pentane ice bath.

Table S1. Energies (in Hartrees) calculated for the singlet and triplet pathways. In addition to the species involved in reactions **[1-3]**, results are provided for the MECP and IM geometries identified in closer examinations of the reaction mechanism. The CCSD(T) total energies include the ZPE correction obtained from the ωB97M-V treatment. Additionally, the relative total energies of the two spin states of each species are provided, as well as the T_1 diagnostic values, $T_1 = |\boldsymbol{t}_1|/\sqrt{N_{elec}}$; the values are all < 0.02, indicating that the single-reference approach is sufficient here.[1]

	ωB97M-V/6-311+G*			rel.	CCSD(T)/def2-qZVP		rel.	
	energy	ZPE	total	(eV)	energy	total	(eV)	T_1
CO($\tilde{X}\,^1\Sigma^+$)	-113.32264820	0.00510898	-113.31753922	0.00	-113.18795255	-113.18284357	0.00	0.005
CO($\tilde{a}\,^3\Pi$)	-113.09592707	0.00415160	-113.09177547	6.14	-112.96561751	-112.96146591	6.02	0.016
MECP(S)	-226.43504966	0.00464648	-226.43040318	---	-226.16851919	-226.16387270	---	0.015
SP(S_1)	-226.38812273	0.00751946	-226.38060326	0.37	-226.12485708	-226.11733762	0.12	0.007
SP(T_0)	-226.40138810	0.00713277	-226.39425533	0.00	-226.12871309	-226.12158032	0.00	0.011
IM(S_1)	-226.50960263	0.01353083	-226.49607180	1.50	-226.23812801	-226.22459718	1.26	0.006
IM(T_0)	-226.56510652	0.01395771	-226.55114864	0.00	-226.28468681	-226.27072910	0.00	0.006
C(^{1}D)	-37.78533003	0.00000000	-37.78533003	1.59	-37.73468982	-37.73468982	1.41	0.002
C(^{3}P)	-37.84369973	0.00000000	-37.84369973	0.00	-37.78650849	-37.78650849	0.00	0.002
CO_2($\tilde{X}\,^1\Sigma_g^+$)	-188.59520444	0.01178996	-188.58341448	0.00	-188.38512061	-188.37333065	0.00	0.005
CO_2($\tilde{a}\,^3B_2$)	-188.42583701	0.00716414	-188.41867287	4.48	-188.21331209	-188.20614795	4.55	0.011
C_2O($\tilde{a}\,^1\Sigma^+$)	-151.22177776	0.00920861	-151.21256915	1.02	-151.02828285	-151.01907424	0.83	0.006
C_2O($\tilde{X}\,^3\Sigma^-$)	-151.25921253	0.00921873	-151.24999380	0.00	-151.05872496	-151.04950623	0.00	0.006
C_3O_2($\tilde{X}\,^1\Sigma_g^+$)	-264.71949842	0.02212149	-264.69737693	0.00	-264.38611376	-264.36399227	0.00	0.006
C_3O_2($\tilde{a}\,^3A$)	-264.59876244	0.01835020	-264.58041224	3.18	-264.26314387	-264.24479367	3.24	0.007

Table S2. ωB97M-V/6-311+G* optimized structures. Cartesian coordinates are given in Å for the polyatomic species whose energies are reported in Table S1.

CO($\tilde{X}\,^1\Sigma^+$)				CO($\tilde{a}\,^3\Pi$)			
C	0.00000	0.00000	-0.64413	C	0.00000	0.00000	-0.68589
O	0.00000	0.00000	0.48256	O	0.00000	0.00000	0.51375
CO_2($\tilde{X}\,^1\Sigma_g^+$)				CO_2($\tilde{a}\,^3B_2$)			
C	0.00000	0.00000	0.00000	C	0.00099	-0.46387	0.00000
O	0.00000	0.00000	-1.15906	O	-1.06162	0.17395	0.00000
O	0.00000	0.00000	1.15906	O	1.06163	0.17395	0.00000
C_2O($\tilde{a}\,^1\Sigma^+$)				C_2O($\tilde{X}\,^3\Sigma^-$)			
C	0.00000	0.00000	-1.41581	C	0.00000	0.00000	-1.41316
C	0.00000	0.00000	-0.05793	C	0.00000	0.00000	-0.05672
O	0.00000	0.00000	1.10530	O	0.00000	0.00000	1.10241
C_3O_2($\tilde{X}\,^1\Sigma_g^+$)				C_3O_2($\tilde{a}\,^3A$)			
C	0.00000	0.00000	0.00000	C	0.02485	-0.34232	-0.25668
C	0.00000	0.00000	1.27359	C	-1.20264	-0.02502	-0.04387
C	0.00000	0.00000	-1.27359	C	1.34711	-0.39248	0.23635
O	0.00000	0.00000	2.42950	O	-2.33971	0.19344	0.08350
O	0.00000	0.00000	-2.42950	O	2.21272	0.37643	-0.03535
SP(S_1)				SP(T_0)			
O	1.48501	-0.15355	-0.00002	O	-1.63003	0.20146	0.07188
C	0.40330	0.32928	0.00004	C	-0.54783	-0.11981	-0.19196
O	-0.99541	0.49069	-0.00002	O	0.62222	-0.38623	0.07726
C	-1.05609	-0.77880	0.00000	C	1.89157	0.36617	-0.00689
IM(S_1)				IM(T_0)			
O	1.44553	-0.02414	0.00000	O	1.82444	-0.00017	0.00000
C	0.29158	0.18272	0.00000	C	0.64164	0.00072	0.00000
O	-0.86688	-0.63747	0.00000	O	-1.82441	0.00024	0.00000
C	-1.06311	0.69943	0.00000	C	-0.64168	-0.00081	0.00000
MECP(S)							
O	-1.45107	0.11285	0.20054				
C	-0.49969	-0.05169	-0.44362				
O	0.85362	-0.54194	0.10490				

Table S3. Summary of ωB97M-V/6-311+G* frequency analyses. For the polyatomic species represented in Table S1, harmonic frequencies and infrared intensities are given in units of cm^{-1} and km mol^{-1}, respectively. Doubly-degenerate vibrational modes are indicated with an asterisk.

	cm^{-1}	km mol^{-1}		cm^{-1}	km mol^{-1}
$CO(\tilde{X}\ ^1\Sigma^+)$	2243	100	$CO(\tilde{a}\ ^3\Pi)$	1822	30
$CO_2(\tilde{X}\ ^1\Sigma_g^+)$	673*	36	$CO_2(\tilde{a}\ ^3B_2)$	628	16
	1394	--		1041	60
	2434	793		1476	9
$C_2O(\tilde{a}\ ^1\Sigma^+)$	353	63	$C_2O(\tilde{X}\ ^3\Sigma^-)$	418*	34
	476	2		1126	18
	1128	32		2085	220
	2086	172			
$C_3O_2(\tilde{X}\ ^1\Sigma_g^+)$	49*	1	$C_3O_2(\tilde{a}\ ^3A)$	84	1
	608*	76		155	6
	609*	--		455	9
	790	--		491	13
	1678	101		574	17
	2305	--		829	27
	2405	3990		1488	2
				1803	264
				2175	754
SP(S_1)	$1477i$	266	SP(T_0)	$1231i$	2382
	339	4		92	8
	425	99		447	22
	742	62		709	110
	1370	21		964	171
	1903	417		2151	454
IM(S_1)	497	18	IM(T_0)	285	--
	513	36		305*	10
	588	139		960	--
	965	71		1824	548
	1303	106		2447	--
	2074	282			
MECP(S)	$1756i$	109			
	$409i$	119			
	346	39			
	602	60			
	1381	88			
	1876	259			

Table S4. Reaction energies, defined as $\Delta E = E(\text{products}) - E(\text{reactants})$, for each of the steps considered in our mechanism. Energies (in eV) are taken from the total energies (including ZPE) given in Table S1.

			ωB97M-V		CCSD(T)	
reactants		products	singlet	triplet	singlet	triplet
CO + CO	→	SP	6.925	0.410	6.758	0.618
SP	→	CO_2 + C	0.323	-0.894	0.254	-1.041
C + CO	→	C_2O	-2.985	-2.415	-2.763	-2.181
C_2O + CO	→	C_3O_2	-4.554	-0.350	-4.410	-0.339

S1. Spectral Assignments

***CO_2*.** The presence of CO_2 as an impurity in the dosing gas used to prepare the samples considered here is evidenced by the appearance of the CO_2 antisymmetric stretch fundamental transition in the pre-excitation spectra in Figure S3. The spectra reported here show a broad feature centered at ca. 2341 cm^{-1} with a sharper feature at 2346 cm^{-1}. The sharper feature lies between two frequencies observed for a pure monolayer of CO_2 on a NaCl(100) surface (see Table S5);[2] the broader feature lies in a range that is in fair agreement with previous reports of IR spectra of CO_2 embedded in CO matrices.[3-8]

Table S5. Summary of spectral features used for kinetic analysis. Observed peak frequencies for CO and CO_2 are obtained from the pre-excitation FTIR spectrum of a sample prepared with $[CO]_0 \sim 300$ ML; that of C_3O_2 was obtained from the final (60000 shots) difference spectrum of the same sample. Uncertainties correspond to the standard error obtained from Lorentzian fits. Where available, literature data for each molecule directly adsorbed to the NaCl(100) surface is also provided. Additionally, frequencies and absorption coefficients used previously to quantify the presence of these molecules in CO matrices are shown.

	obs. freq. (cm^{-1})	FWHM (cm^{-1})	NaCl(100) (cm^{-1})	lit. freq. (cm^{-1})	$\bar{\sigma}_g \times 10^{16}$ (cm molecule^{-1})
CO	2138.534(7)<br>2143.02(1)	0.96(2)<br>0.63(4)	2138.51[9]<br>2142.54[9]	2136[7]	0.11[10]
C_3O_2	2242.60(7)	6.6(3)	--	2242[7]	3.6[11]
CO_2	2340.7(1)<br>2346.77(1)	5.3(5)<br>0.60(3)	2340.1[2]<br>2349.0[2]	2346[7]	1.4[12]

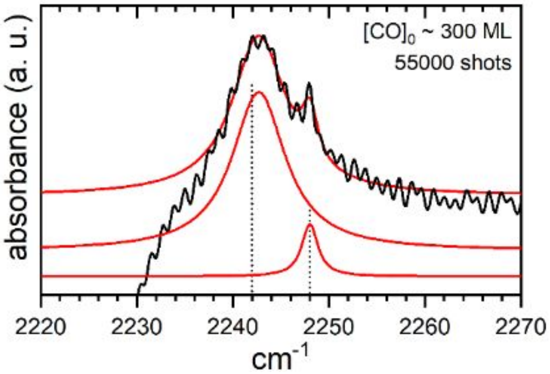

Figure S4. Lineshape of the shot-dependent feature at ca. 2250 cm^{-1}. The difference absorption spectrum for a $[CO]_0 \sim 300$ ML sample exposed to 55000 laser shots is shown in black. Fitting this to a two-peak Lorentzian lineshape results in the red traces, where the component peaks are also shown. Dashed lines indicate previously reported absorption frequencies assigned to C_3O_2 and C_3O in CO matrices.[7]

***C_3O_2, C_3O*.** As shown in Figure S4, the shot-dependent feature at ca. 2250 cm^{-1} shows a lineshape consistent with two underlying transitions at ~2242 and 2248 cm^{-1}. The infrared absorption spectrum of C_3O_2 diluted in a CO matrix shows a strong absorption at ~2242 cm^{-1}, as well as a weaker combination band at ~2400 cm^{-1};[4] these features have been clearly identified in CO ices following irradiation with protons or UV photons.[3-5] Though fairly weak, there is some evidence for the ca. 2400 cm^{-1} combination band in our

spectra (Fig. S5, blue area). We can thus confidently assign at least the 2242 cm^{-1} component as arising from formation of C_3O_2 in our sample.

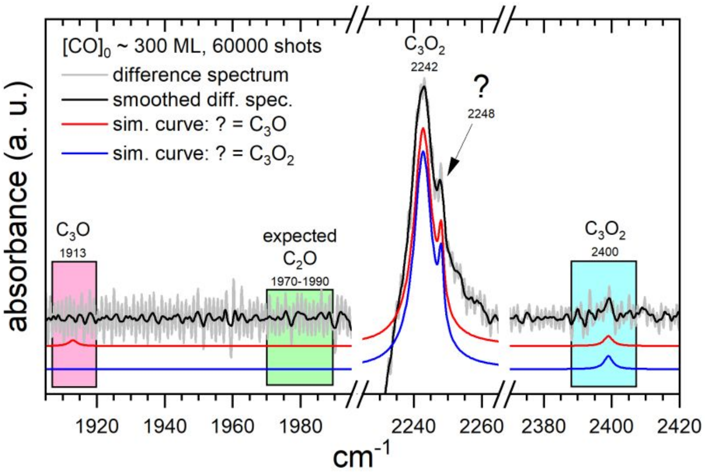

Figure S5. Demonstration of the absence of spectral evidence for formation of C_3O and C_2O. The difference absorption spectrum for a ~300 ML sample is shown in gray, with a smoothed version shown in black for clarity. The red trace shows the spectrum simulated by assuming that the side-peak at 2248 cm^{-1} represents the formation of C_3O, leading to the appearance of a weak feature at 1913 cm^{-1} that is not seen experimentally. The blue trace is a spectrum simulated with the assumption that the area under both ~2250 cm^{-1} peak components contributes to the calculation of [C_3O_2]. Additionally, the C_2O region of the spectrum is highlighted in green.

In previous reports of C_3O_2 formation in CO ices, the lineshape of the ca. 2250 cm^{-1} band observed here always shows some degree of asymmetry, with some reports achieving sufficient resolution to reveal a side-peak at ~2248 cm^{-1} consistent with the weaker component of Fig. S4. In some cases, the evolution of this side-band was noted to correlate with the increase of a lower-intensity transition below 1950 cm^{-1} assigned to C_3O.[3, 7] However, as noted by Trottier *et al.*,[5] it is also possible that this side-peak corresponds to a matrix perturbation of the C_3O_2 band.

Figure S5 shows an experimental post-excitation difference spectrum in the regions where C_3O_2 and C_3O are known to absorb. If the 2248 cm^{-1} side-band corresponds to C_3O, the absorption coefficients reported by Jamieson and coworkers[7] may be used to simulate the intensity of the ca. 1913 cm^{-1} transition of C_3O. As shown in the red trace of Figure S5 (pink area), this would likely be below our detection threshold. However, we may also simulate the expected intensity for the C_3O_2 combination band at ca. 2400 cm^{-1}; if we consider the area under the 2248 cm^{-1} feature to contribute to the $\tilde{A}_p$ value used in calculating [C_3O_2] (Section S2a), we find better agreement for this weak transition (blue curve, blue area in Fig. S5).

Additionally, we note that the formation of C_3O would occur through the reaction

$$C + C_2O \rightarrow C_3O.$$

For this to take place in our samples, the reactive products of reactions **[1-2]** must be generated in close proximity, or diffuse together before reacting with a CO molecule. Previous studies of CO/NaCl(100) have shown that the highest-energy adsorbates produced through VEP are quite dilute,[13] so the likelihood of two nearby CO molecules achieving sufficient excitation to proceed through **[1]** is quite low. Because of this low density of reaction centers, as well as the limited diffusion inherent in a 7K ice, we do not consider C_3O as a significant contributor to our observed spectra, and we take the dominant carrier of the ca. 2250 cm^{-1} signal in our spectra to be carbon suboxide.

C_2O. In high-energy irradiation of CO ices, the formation of C_2O is typically monitored through its absorption at ca. 1980-1990 cm^{-1}.[7] This region where C_2O would absorb is highlighted in Figure S5 (green area), and shows that this molecule is not accumulated in sufficient quantities to observe.

S2. Quantitative Analysis of FTIR Spectra

a. Calculation of Column Densities

The integrated absorbances $\tilde{A}_p$ of the spectral features summarized in Table S5 are used to extract column densities of CO, CO_2, and C_3O_2 from the post-excitation FTIR spectra produced by the excite-probe procedure. The column densities, in units of monolayer (ML), are calculated by

$$[X] = \frac{\ln(10) \cos\theta}{N\bar{\sigma}_g \rho_2} \tilde{A}_p,$$

where $\bar{\sigma}_g$ is the gas-phase integrated absorption coefficient (in cm $molecule^{-1}$, see Table S5), $N = 1$ is the number of surfaces probed, and $\theta = 45°$ is the angle of incidence of the FTIR light source. The 2D density associated with a single layer within the α-CO crystal is given by $\rho_2 = 2/a_0^2 \approx 6.27 \times 10^{14}$ molecules cm^{-2}, where a_0 is the lattice constant for α-CO at 8K.[14]

For CO, the integrated absorbance is taken to be the sum of the LO and TO peak areas, obtained by numerically integrating the background-subtracted spectra in the 2135-2145 cm^{-1} region. The CO_2 column density was obtained in a similar manner by taking the numerical integral of the 2330-2355 cm^{-1} spectral region, so that the results include contributions from both components of the observed band. The absolute

CO_2 column densities obtained in this manner reflect not only that formed due to interaction with the laser, but also the CO_2 initially present in the samples, as well as the roughly linear increase arising from background adsorption. The reported column densities in Figure 2 have been corrected for these effects (see Fig. S6).

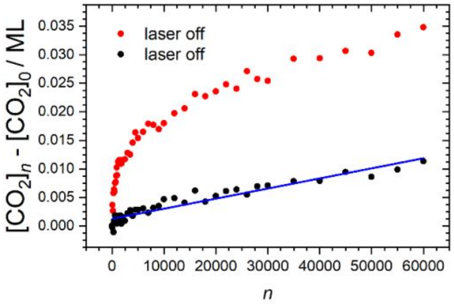

Figure S6. Growth of CO_2 column density over the time scale of the excite-probe procedure. The excite-probe program was applied to a ~150 ML sample of CO with (red) and without (black) laser excitation, and column densities were calculated as described in Section S2a to assess the increase in CO_2 that occurs solely due to adsorption of residual gas in the UHV chamber. This linear increase is indicated by the blue line, which was subtracted from all CO_2 column densities to correct for this background signal.

For C_3O_2, the overlap of the spectral feature with the CO phonon band complicated evaluation of the integrated absorbance; results were calculated from the ~2240-2280 cm^{-1} range of the difference spectra (where the spectrum of the initial sample is subtracted from the post-excitation result). This range was selected to minimize contributions from the CO phonon side-band, but may not fully account for the integrated absorbance arising from carbon suboxide.

b. Yield Analysis of Concentration Data

As can be seen from Figure 2, the extent of product formation is far too small to fully explain the observed CO depletion. Defining the yield of CO depletion as $\Delta[\mathrm{CO}]/[\mathrm{CO}]_0$ where $\Delta[X] = |[X]_{60000} - [X]_0|$, we find that ~33, 53, and 58% of the initial CO coverage was lost after 60000 laser shots for samples prepared with $[\mathrm{CO}]_0$ ~ 150, 300, and 500 layers, respectively. To determine the portion of this CO loss that can be attributed to chemical transformations, we consider CO_2 and C_3O_2 to be the only products and CO the only reactant, so that the net reaction is given by

$$4\mathrm{CO} \rightarrow \mathrm{CO_2} + \mathrm{C_3O_2}.$$

With this, and noting that in all three cases the calculated amount of CO_2 formed is greater than or equal to C_3O_2, the maximum amount of CO consumed by chemical reactions can be estimated as $4 \times \Delta[\mathrm{CO_2}]$. Dividing by $\Delta[\mathrm{CO}]$ shows that for all samples, less than 0.2% of the total CO lost can be accounted for by production of CO_2 and C_3O_2. These percentages, as well as the absolute changes in concentrations and percent product yields (given by $4\Delta[X]/[\mathrm{CO}]_0$), are provided in Table S6.

Assessing the dependence on thickness is complicated by the fact that the thickest sample was prepared with natural abundance CO, whereas the two thinner samples were prepared using isotopically pure $^{12}C^{16}O$; the enhanced presence of isotopic impurities likely impacts the pooling dynamics in the ca. 500 ML sample.[15] However, some trends may be identified, in particular when the column densities are converted to more intuitive units. The number of layers in the sample (given by the sum of the column densities of all three species) may be multiplied by the thickness of a layer of α-CO (given by the ratio ρ_2/ρ_3 with ρ_3 = 2.22×10^{22} molecules cm^{-3}) to yield the total thickness of the sample (Fig. S7a).[14] Column densities (molecules cm^{-2}, obtained by multiplying the data in Fig. 2 by ρ_2) may then be converted to concentrations (molecules cm^{-3}) by dividing by this thickness; the volumetric concentrations are used to obtain the fractional composition of our sample over the course of the excite-probe procedure (Fig. S7b).

Considering these results, we note that while increasingly thick samples show larger percent depletion of CO (Table S6), the overall change in sample composition decreases. In all three cases the product formation saturates when the photoproducts constitute ca. 0.02% of the total sample, though this slightly decreases as sample thickness increases.

Table S6. Initial [CO] and changes in concentrations for CO, CO_2, and C_3O_2 from the data represented in Figure 2. The maximum percentage of CO depletion that can be attributed to reactions is calculated from the ratio $4\Delta[\mathrm{CO_2}]/\Delta[\mathrm{CO}]$, whereas the percent yield for formation of product X is obtained from $4\Delta[X]/[\mathrm{CO}]_0$.

$[\mathrm{CO}]_0$ / ML	153	316	494
$\Delta[\mathrm{CO}]$ / ML	51	166	288
$\Delta[\mathrm{CO_2}]$ / ML	0.024	0.030	0.029
$\Delta[\mathrm{C_3O_2}]$ / ML	0.024	0.029	0.024
net % CO desorption	33	53	58
max % CO lost to reactions	0.190	0.071	0.040
% yield CO_2	0.063	0.037	0.024
% yield C_3O_2	0.062	0.036	0.019

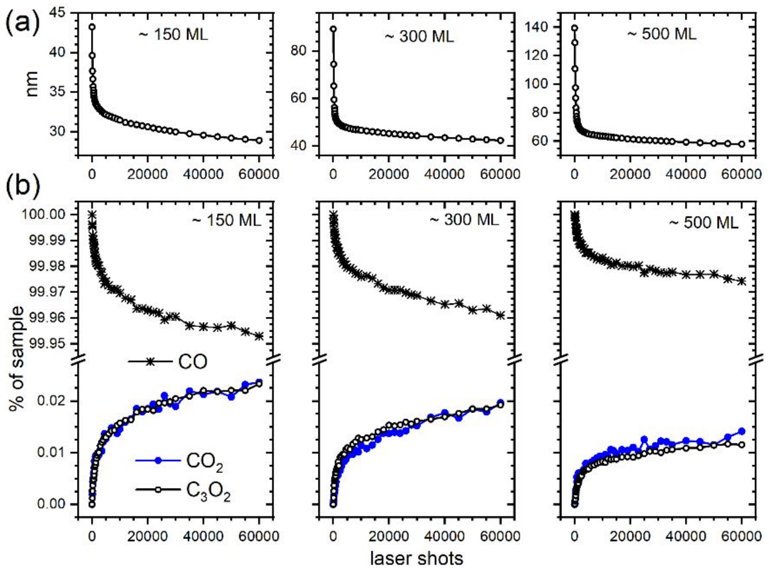

Figure S7. Alternative representation of the data in Figure 2. (a) Sample thickness as a function of laser shots. The thickness is calculated from the ML data by assuming a layer thickness representative of pure α-CO. (b) Percentage of total sample represented by CO, CO_2, and C_3O_2, neglecting any other species.

S3. Comparison of Different Levels of Theory

The geometries of the reactants, TS, and products for reactions **[1-3]** were also obtained at the B3LYP/aug-cc-pVTZ and PTPSS-D_3/6-311+G* levels of theory. These results are compared to the ωB97M-V/6-311+G* results in Figure S8a, showing similar energetic pictures for reactions **[1-3]** as is given in Figure 3 of the main text.

As noted in the main text, the calculated barrier heights for reaction **[1]** in our work show that the SP(S_1) geometry is lower in energy than the CO_2 + C products, in contrast to the results of Barreto and coworkers.[16] To investigate this discrepancy, repeated calculations at the MP2/aug-cc-pVTZ level of theory were performed to obtain the geometries and frequencies of all species involved in reaction **[1]**, followed by single-point CCSD(T)/aug-cc-pVQZ calculations; this was the approach used in the prior work. The results of these theoretical methods, as well as the B3LYP results mentioned above, are shown in Figure S8b. Only the MP2 level of theory gives a singlet SP energy greater than that of the products to **[1]**; the CCSD(T) energies of the MP2 geometries give a similar energetic picture to that obtained from DFT for the singlet pathway, although the triplet pathway shows that the conversion to CO_2 + C is effectively barrierless. Further, an IRC calculation on the MP2/aug-cc-pVTZ singlet SP shows the same behavior represented in Figure 4B, indicating that for all model chemistries used here, the SP(S_1) geometry is in actuality a transition state to the formation of IM(S_1) from 2CO molecules.

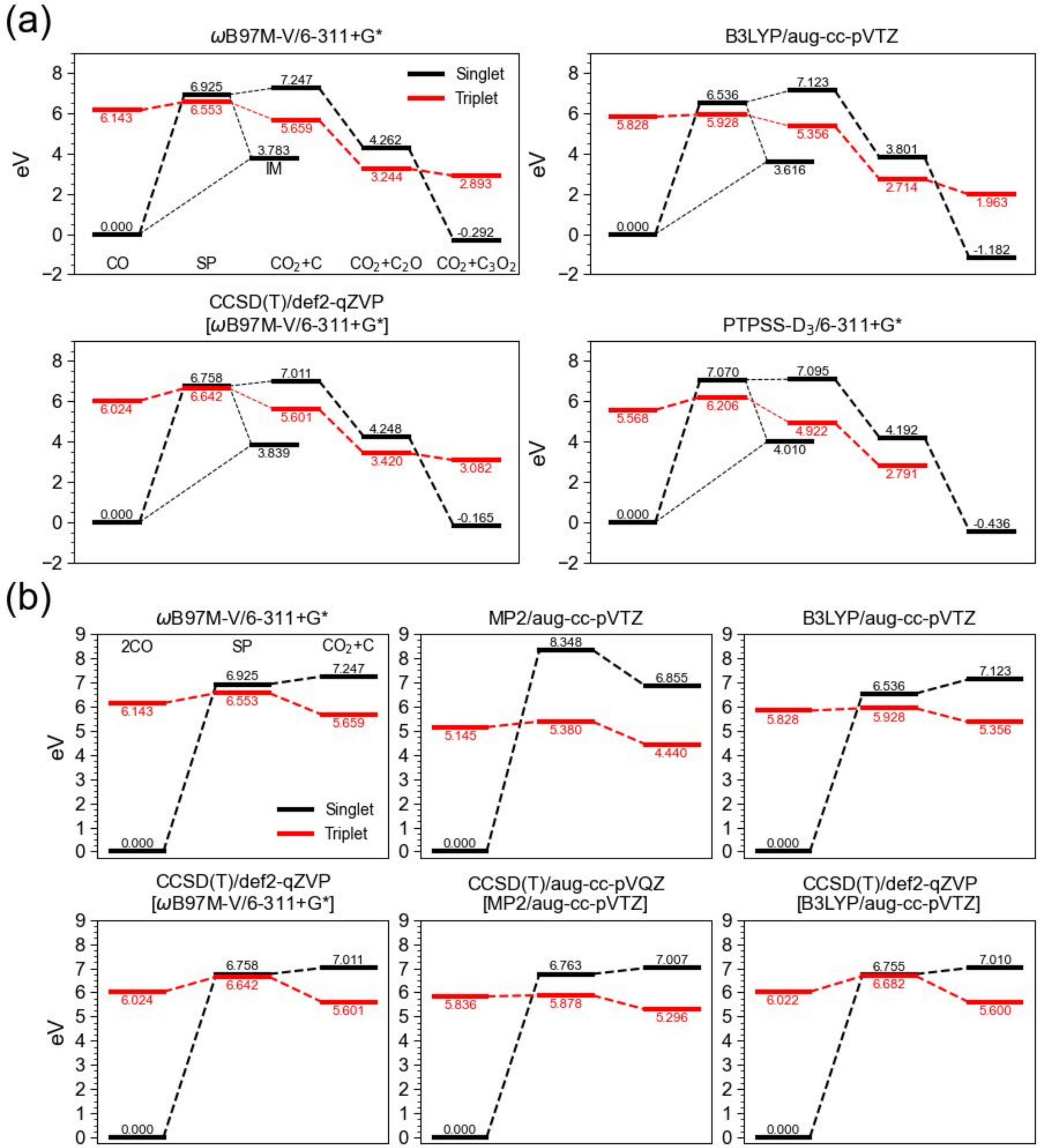

Figure S8. Comparison of different levels of theory. Potential energy diagrams for the singlet (black) and triplet (red) pathways are shown. For CCSD(T) calculations, the method used to obtain geometries and ZPE corrections is shown in brackets. Energies of each state are given in units of eV relative to the 4CO reactant well. **(a)** Overall reaction given by **[1-3]**. Thinner dashed lines indicate rearrangement, whereas the thicker dashed lines indicated addition of a CO molecule. **(b)** Reaction **[1]** portion of the mechanism.

S4. Further Calculations Regarding SP(S_1) and IM(S_1)

a. Stationary State Calculations

To connect SP(S_1) to the well-behaved SP(T_0), a spin-flip time-dependent DFT (SF-TDDFT) calculation was performed at the SP(T_0) equilibrium geometry using our chosen ωB97M-V/6-311+G* method.[17] A subsequent TS optimization of the lowest-lying singlet excited state of SP(T_0) leads to the SP(S_1) geometry. Thus, while the IRC calculation suggests that SP(S_1) may not represent the true transition state for reaction **[1]** on the singlet surface, it does correspond to an electronically excited state of a well-behaved TS for **[1]** on the triplet surface.

To determine whether a second transition state connects IM(S_1) to the desired products of **[1]**, an additional ωB97M-V/6-311+G* FSM calculation was performed, yielding the results in Figure S9. This shows that there is no singlet geometry that can serve as a saddle point connecting the reactants and products of reaction **[1]**, so that the purely singlet pathway is inconsistent with our observation of CO_2 and C_3O_2 production. A similar FSM calculation was performed to connect IM(S_1) to the singlet 2CO reactants, showing that IM(S_1) → 2CO decomposition is a barrierless process.

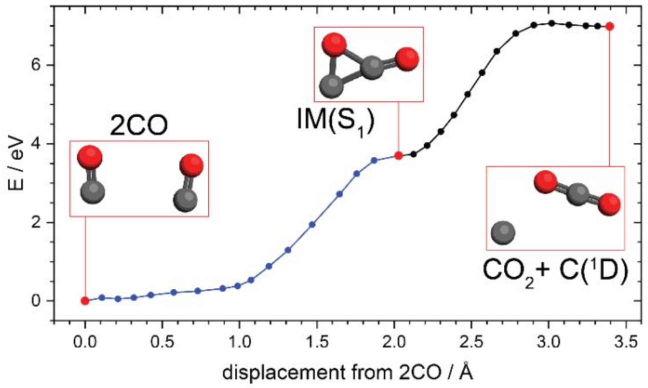

Figure S9. FSM calculations regarding IM(S_1). The FSM results for (blue) 2CO → IM(S_1) and (black) IM(S_1) → CO_2 + C(^{1}D) are shown. Energies do not include ZPE effects and were obtained at the ωB97M-V/6-311+G* level of theory. The "reactant" and "product" geometries used as inputs for the FSM calculations are also shown.

One could also imagine that the IM(S_1) geometry leads to formation of cyclic C_2O, *c*-C_2O, which is identified as a stable isomer on the singlet surface. This could occur through dissociation, IM(S_1) → *c*-C_2O + O, and the remaining O atom would then react with a CO molecule to form CO_2. Alternatively, the cyclic structure could be formed by direct reaction of IM(S_1) with CO, IM(S_1) + CO → *c*-C_2O + CO_2. These

possibilities were explored using our ωB97M-V/6-311+G* framework, yielding the energy diagram of Figure S10. In both cases formation of the cyclic structure requires surmounting a reaction barrier; given the results shown in Figure S9 it seems far more likely that IM(S_1) dissociates to form 2CO molecules, rather than proceeding to products through a cyclic intermediate.

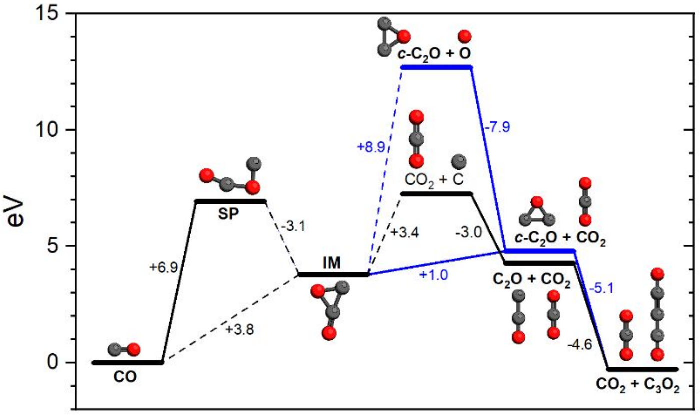

Figure S10. Potential energy diagram for the singlet reaction pathways involving *c*-C_2O. Energies are obtained at the ωB97M-V/6-311+G* level of theory and include ZPE corrections. Solid lines are used to indicate addition of a CO molecule, and dashed lines indicate rearrangement. All energies are given in units of eV and represent the energy difference in going from the left to the right state.

b. Molecular Dynamics Simulations

While the calculations summarized above indicate that the lowest-energy singlet pathway does not provide a clear connection between IM(S_1) and products, it should be noted that IM(S_1) formed from SP(S_1) will at first possess considerable energy. This could provide access to alternative, higher-energy pathways capable of forming the CO_2 + C(^{1}D) products. To determine this possibility, Born-Oppenheimer molecular dynamics (BOMD) simulations were performed in Q-Chem using the ωB97M-V/6-311+G* framework. These trajectories propagated the IM(S_1) geometry with an initial kinetic energy (KE) given by one of the two values illustrated in Figure S11. From this diagram it can be seen that the lower-KE choice (KE_1) represents formation of IM(S_1) at the ZPE of SP(S_1), which is nearly degenerate with the potential energy of the CO_2 + C(^{1}D) products. We also consider a higher-KE choice (KE_2), which would correspond to formation of vibrationally-excited SP(S_1) formed from CO molecules at the uppermost energy levels known to be populated through VEP. For both KE choices, fifty sets of initial velocities were generated which randomly distributed this kinetic energy along the six normal modes of IM(S_1) illustrated in Fig. S11.

Dynamics were simulated for 500 time steps of duration 20 a.u. (0.484 *f*s), using the microcanonical (*NVE*) ensemble so that the total energy remains constant.

In all cases, the structure dissociated into 2 CO fragments moving away from each other. This can be seen by the representative atom-atom displacement plots in Fig. S12-S13, where at long times, the only two atom-atom separations within bonding tolerances are the two separated CO monomers (O_1-C_2 / O_3-C_4). All other displacements diverge, representing increasing separation between the CO centers-of-mass (COMs). Defining t_{sep} as the first time point where the separation between COMs of the CO fragments is > 3 Å, we can construct the histograms in Figure S14, showing that even when given sufficient energy to exceed the barrier to form CO_2 + C(^{1}D), trajectories initialized on the singlet pathway rapidly lead back to 2CO molecules.

The constant-energy conditions used to propagate the trajectories represented in Figures S12-S13 differ from the experimental situation, where the molecular system is embedded in a 7K environment that can serve as a heat sink. Thus, by assuming energy conservation, the BOMD simulations performed here should provide an enhanced probability of observing conversion to CO_2 + C(^{1}D), particularly in the E_2 simulations where IM(S_1) is initialized with sufficient energy to energetically access this geometry. Thus, the results here clearly show the unlikelihood of forming CO_2 on the singlet pathway within our theoretical framework.

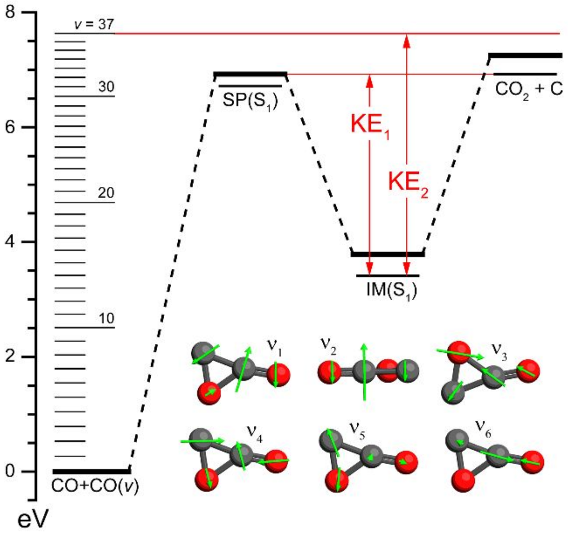

Figure S11. Initial conditions used in BOMD simulations of IM(S_1). Kinetic energies as defined by the red arrows were randomly distributed along the six normal modes of IM(S_1) pictured here. Energies are obtained at the ωB9M-V/6-311+G* level of theory. Thicker lines indicate the energies with ZPE corrections; the non-ZPE-corrected energies are shown as the thinner lines beneath each ZPE-corrected level.

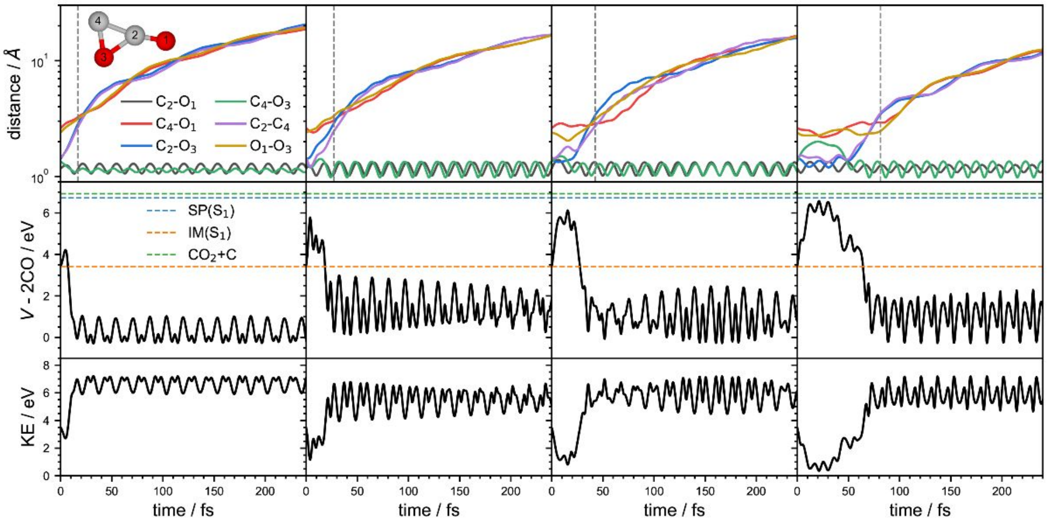

Figure S12. Representative BOMD trajectories for $IM(S_1)$ with an initial kinetic energy of 3.510 eV. The qualitative behavior shown here is also observed for the 46 other trajectories. From top to bottom, each column shows the atom-atom displacements (see inset for numbering convention), potential energy V relative to 2CO, and kinetic energy for each trajectory. Energies of relevant species are indicated in the potential energy plots.

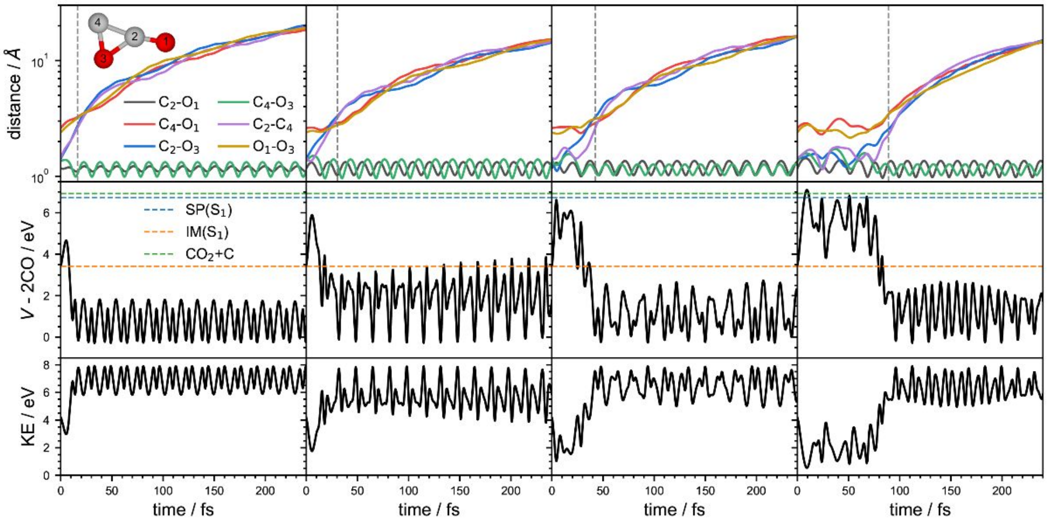

Figure S13. Representative BOMD trajectories for $IM(S_1)$ with an initial kinetic energy of 4.223 eV. The qualitative behavior shown here is also observed for the 46 other trajectories. From top to bottom, each column shows the atom-atom displacements (see inset for numbering convention), potential energy V relative to 2CO, and kinetic energy for each trajectory. Energies of relevant species are indicated in the potential energy plots.

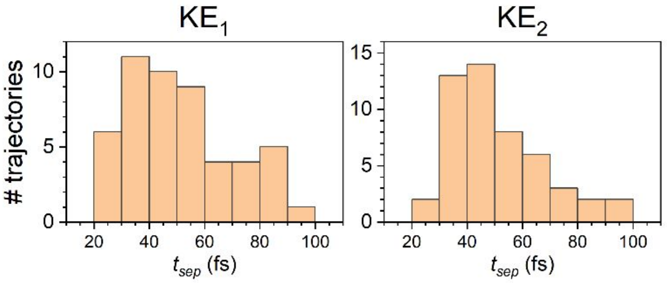

Figure S14. Histograms of the dissociation time for all BOMD trajectories. The dissociation time t_{sep} is defined as the earliest time at which the distance between the centers-of-mass of the 2CO fragments exceeds 3 Å.

S5. Curve-Crossing in Reaction [1]

The reaction pathway calculations represented by Fig. 3 suggest a crossing of the singlet and triplet surfaces of reaction **[1]** in the vicinity of the transition state. To study this in more detail, the structures generated in the triplet IRC (Fig. 4A) were used to simulate geometries sampled at even intervals along the reaction coordinate of **[1]**. Single-point CCSD(T)/def2-qZVP calculations on these geometries in the singlet and triplet states yield the energy diagram of Figure S15a, which represents the singlet and triplet diabatic surfaces through the conversion of 2CO to CO_2 + C. The crossing point for these non-optimized surfaces is found to occur shortly before the triplet transition state geometry; this was used as an initial structure to determine the minimum-energy crossing point (MECP) along the reaction pathway of **[1]**.

The MECP optimization was performed at the ωB97M-V/6-311+G* level of theory, where the branching-plane method was used to identify the crossing point for the (reference) singlet state with the first SF-TDDFT triplet state.[18] The CCSD(T)/def2-qZVP energy of this species, including ZPE correction, is shown in Figure S15b. As shown in Fig. S16, the MECP geometry largely resembles SP(S_1), but has a torsional angle that is more consistent with that of a CO dimer in an α-CO crystal (Table S7).

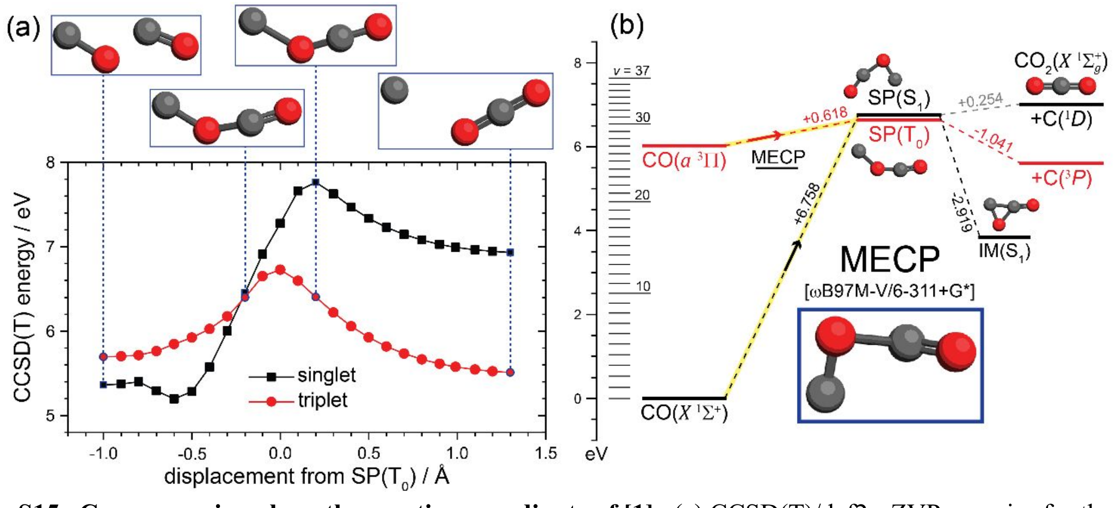

Figure S15. Curve crossing along the reaction coordinate of [1]. (a) CCSD(T)/def2-qZVP energies for the singlet and triplet states throughout reaction **[1]**, where some key geometries have been noted. Energies are given relative to the CCSD(T)/def2-qZVP energy of 2CO($\tilde{X}\ ^1\Sigma^+$) (without ZPE correction). (b) Expanded view of the **[1]** portion of Figure 3, showing the ZPE-corrected MECP energy.

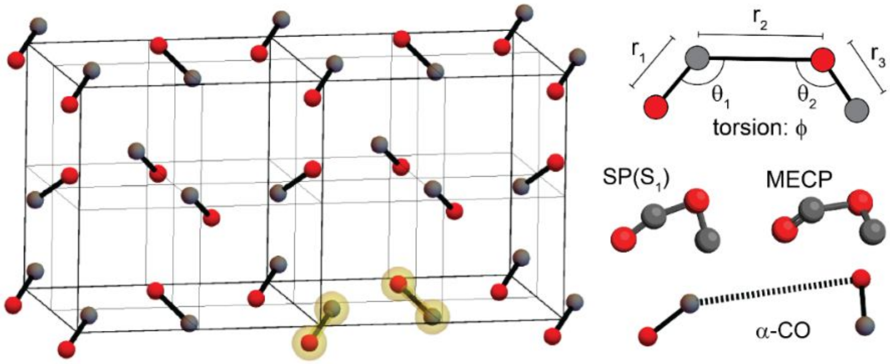

Figure S16. Structure of a CO dimer within the α-CO crystal lattice, and comparison to the dimer structures identified in exploration of the reaction pathway. The crystal structure of α-CO, assuming a CO bond distance of 1.128 Å and a lattice constant of 5.646 Å, is shown on the left.[19] The "dimer" structure formed by considering the two highlighted molecules is shown in the bottom-right, alongside the ωB97M-V/6-311+G* geometries of SP(S_1) and the MECP for **[1]** (middle-right). All dimer structures may be described in terms of the six geometrical parameters shown in the top-right; these parameters are presented in Table S7, showing that the three angular coordinates are strikingly similar between the α-CO and MECP cases.

Table S7. Geometrical parameters for the CO dimer structures considered here. Parameters as defined in Figure S16 are shown for the singlet/triplet SP, the ωB97M-V/6-311+G* MECP, as well as a CO dimer in α-CO. The parameters for the crystal structure are obtained using the same parameters specified in Fig. S16 – i.e. an equilibrium CO bond distance of r_e 1.128 Å and a lattice constant of 5.646 Å – where the average bond length is given by $\langle \phi_n | r | \phi_n \rangle$ obtained from the Morse oscillator wavefunctions associated with the ground singlet state of CO.[20]

	r_1 (Å)	r_2 (Å)	r_3 (Å)	θ_1 (°)	θ_2 (°)	ϕ (°)
SP(S_1)	1.185	1.408	1.271	169	86	-0.02
SP(T_0)	1.159	1.230	1.478	154	134	-81
MECP	1.161	1.540	1.249	125	90	77
α-CO (v = 0)	1.132	3.560	1.132	144	84	65
α-CO (v = 30)	1.132	3.436	1.442	143	85	65

S6. Intersystem Crossing Rates

a. Fermi's Golden Rule Model

We consider non-radiative transitions of the type

$$A(i,\vec{v}) \rightsquigarrow A(f,\vec{w}) \quad \Delta E = E^{i}_{\vec{v}} - E^{f}_{\vec{w}} \#(S1)$$

where i and f are two electronic states of molecule A with differing spins. The N-dimensional vectors $\vec{v}$ and $\vec{w}$ contain the N quantum numbers specifying the vibrational levels of the initial and final states. We will restrict our considerations to exoergic transitions, so that ΔE as defined in Eq. (S1) is always positive. We consider that the energy released by Eq. (S1) is absorbed by the phonons of the surrounding CO crystal, as discussed in Section S6b.

Within the Born-Oppenheimer approximation, the initial and final states may be expressed as

$$|\Psi_i\rangle = |\psi_i\rangle|\vec{v}\rangle \quad |\Psi_f\rangle = |\psi_f\rangle|\vec{w}\rangle\#(S2)$$

where $|\psi_{i,f}\rangle$ represent the solutions to the standard Born-Oppenheimer electronic Hamiltonian and the nuclear coordinates are encapsulated by $|\vec{v}\rangle$ and $|\vec{w}\rangle$. In the ISC processes described by Eq. (S1), these states are coupled by the spin-orbit coupling (SOC) operator, $\hat{H}_{SO}$;[21] in the Franck-Condon (FC) limit, where this operator purely acts on the electronic wavefunctions, Fermi's Golden Rule gives the rate of transition between these states as

$$k^{FC}_{if} = \frac{2\pi}{\hbar}\left|\left\langle\psi_f \,\middle|\, \hat{H}_{SO} \,\middle|\, \psi_i\right\rangle\right|^2 |\langle\vec{w}\,|\,\vec{v}\rangle|^2 \rho(\Delta E).\#(S3)$$

Here, $\rho(\Delta E)$ replaces the typically-employed delta function, due to the ability of the CO phonon bath to account for the energy discrepancy in Eq. (S1) (see Section S6b).

Given the observation that the singlet and triplet surfaces in Fig. 3 cross, it seems plausible that the SOCs of interest in the current system will depend on the nuclear configuration. We therefore will also consider a spin-vibronic (SV) mechanism for ISC.[22] In this framework, the vibronic states given in Eq. (S2) are coupled through the spin-vibronic Hamiltonian, $\hat{H}_{SV}$, expressed as an expansion of the purely-electronic $\hat{H}_{SO}$ around a reference geometry, $\vec{x}_i$,

$$\hat{H}_{SV} = \hat{H}_{SO} + \sum_{\alpha} \left(\frac{\partial \hat{H}_{SO}}{\partial x_\alpha} \right)_{\vec{x}_i} x_\alpha$$

where x_α is the αth normal coordinate of A. With this, the SV-ISC rate is

$$k_{if}^{SV} = \frac{2\pi}{\hbar} \left| \langle \Psi_f \mid \hat{H}_{SV} \mid \Psi_i \rangle \right|^2 \rho(\Delta E) \#(S4)$$

where the SV matrix elements are given by

$$\langle \Psi_f \mid \hat{H}_{SV} \mid \Psi_i \rangle = \langle \psi_f \mid \hat{H}_{SO} \mid \psi_i \rangle_{\vec{x}_i} \langle \vec{w} \mid \vec{v} \rangle + \sum_{\alpha} b_\alpha^i \langle \vec{w} \mid x_\alpha \mid \vec{v} \rangle \quad \text{with} \quad b_\alpha^i = \left(\frac{\partial \langle \psi_f \mid \hat{H}_{SO} \mid \psi_i \rangle}{\partial x_\alpha} \right)_{\vec{x}_i} . \#(S5)$$

From the state-to-state rates given by Eqs. (S3-S4), the total FC- and SV-ISC rates for a particular initial state *i* are then calculated by summing over the manifold of final states *f*,

$$k_i^{FC} = \sum_f k_{if}^{FC} \qquad k_i^{SV} = \sum_f k_{if}^{SV} . \#(S6)$$

b. Treatment of the Phonon Density of States

As noted above, we consider the surrounding CO matrix and its vibrational density of states (DOS) to serve as a means of ensuring energy conservation. To do this, rather than using a delta function in our rate expressions, we use $\rho(\Delta E)$, the phonon DOS of the surrounding crystal at the energy difference between the initial and final vibronic levels of A. Given the low temperature of our system, we restrict our considerations to exothermic processes where ΔE as defined in Eq. (S1) is strictly positive.

Previous studies of the phonon DOS for α-CO indicate that the phonon spectrum is highly structured and cuts off above 150 cm^{-1}.[23] For simplicity, we use a three-dimensional Debye DOS in Eqs. (S3-S4), with a cutoff frequency of $\omega_D = 200$ cm^{-1}.

$$\rho(E) = \begin{cases} \frac{3E^2}{(hc\omega_D)^3} & E \leq hc\omega_D \\ 0 & E > hc\omega_D \end{cases}$$

Calculations of the rates discussed below were also tested using the NaCl(100) vibrational DOS reported previously[13] as well as an approximation to the α-CO phonon DOS;[23] the resultant rates are the same order of magnitude of the ones presented here.

S7. ISC in a CO Dimer

a. SP(S_1) ⇝ SP(T_0)

Given the singlet/triplet curve crossing identified for a CO dimer geometry, we will apply the ISC mechanisms discussed above to transitions of the form

$$\mathrm{SP}(\mathrm{S}_1,\vec{v}) \rightsquigarrow \mathrm{SP}(\mathrm{T}_0,\vec{w}).$$

The possibility for transitions involving other dimer structures is considered in Section S7b.

For evaluation of the nuclear integrals, we express the vibrational wavefunctions for levels in the triplet $\varphi_{\vec{w}}^{T}$ and singlet $\varphi_{\vec{v}}^{S}$ electronic states as products of 1D harmonic oscillators,

$$\varphi_{\vec{w}}^{T}(\vec{x}^{T}) = \prod_{\alpha} \phi_{w_\alpha}^{T}(x_\alpha^{T}) \qquad \varphi_{\vec{v}}^{S}(\vec{x}^{S}) = \prod_{\alpha} \phi_{v_\alpha}^{S}(x_\alpha^{S})$$

where x_α^T and x_α^S are the normal coordinates of the triplet and singlet state, respectively, and the vectors $\vec{x}^{T,S}$ represent displacements expressed in the basis of the corresponding state's normal coordinates. The 1D wavefunctions are taken to be

$$\phi_{v_\alpha}^{i}(x) = \frac{1}{\sqrt{2^{v_\alpha} v_\alpha!}} \left(\frac{\mu_\alpha^i c \omega_\alpha^i}{\pi h}\right)^{1/4} \exp\left(-\frac{\mu_\alpha^i c \omega_\alpha^i x^2}{2h}\right) H_{v_\alpha}\left(\sqrt{\mu_\alpha^i c \omega_\alpha^i / h}\, x\right) \quad [i = S,T] \# (S7)$$

where μ_α^i and ω_α^i are the reduced mass and frequency (in cm^{-1}) associated with mode α in state i.

We note that evaluation of the nuclear integrals involved in the SV- and FC-ISC rates requires one to express the vibrational wavefunctions given above in terms of the same set of coordinates. However, the frequency analyses of SP(S_1) and SP(T_0) show that the normal coordinates $\{x_\alpha^T\}$ and $\{x_\alpha^S\}$ are not the same in both cases. The most rigorous treatment would be to use a Duschinsky transformation[24] on the singlet state normal coordinates to obtain these as linear combinations of those of the triplet state (or vice versa); this prevents the expression of the nuclear integrals as products of one-dimensional results.

To avoid this difficulty, we instead invoke a parallel approximation. We assume that the normal modes of the singlet and triplet states are similar enough that the same coordinates can be used to express vibronic wavefunctions in both states, so that the normal coordinates of the singlet state are given by $x_\alpha^S = x_\alpha^T + \Delta x_\alpha$.

Figure S17 shows the mode-matching convention used; as vibrational modes with imaginary frequencies are not appropriate for treatment with a harmonic oscillator model, we neglect ν_1 in our rate evaluations. We then calculate the coordinate shifts Δx_α, which are obtained from the equilibrium geometries $\vec{x}_T$ and $\vec{x}_S$ as well as the (normalized) atomic displacement vector associated with mode ν_α, $\vec{q}^{\,T}_\alpha$, by

$$\Delta x_\alpha = \left(\vec{x}_S - \vec{x}_T\right) \cdot \vec{q}^{\,T}_\alpha.$$

With these approximations we obtain our final expressions for our vibrational wavefunctions,

$$\varphi^T_{\vec{w}}(\vec{x}) = \prod_{\alpha=2}^{6} \phi^T_{w_\alpha}(x_\alpha) \qquad \varphi^S_{\vec{v}}(\vec{x}) = \prod_{\alpha=2}^{6} \phi^S_{v_\alpha}(x_\alpha - \Delta x_\alpha)$$

where the ϕ^i_v are given by Eq. (S7). The energies of these states are taken to be

$E^T_{\vec{w}} = hc\sum_{\alpha=2}^{6}\omega^T_\alpha w_\alpha \qquad E^S_{\vec{v}} = \epsilon_S + hc\sum_{\alpha=2}^{6}\omega^S_\alpha v_\alpha$ where ϵ_S/hc = 931.2 cm^{-1} is the energy difference between the ZPEs of SP(S_1) and SP(T_0) obtained from our CCSD(T) results in Table S1. Mode-specific parameters were obtained by our computational work and are summarized in Table S8.

We then express the SV matrix element $\langle \Psi_i | \hat{H}_{SV} | \Psi_f \rangle$ which couples the $\vec{v} = (v_2,v_3,v_4,v_5,v_6)$ level of SP(S_1) to the $\vec{w} = (w_2,w_3,w_4,w_5,w_6)$ level of SP(T_0) as

$$\langle \Psi_i | \hat{H}_{SV} | \Psi_f \rangle = \langle \psi_S | \hat{H}_{SO} | \psi_T \rangle_i \left\{ \prod_{\alpha=2}^{6} \int_{-\infty}^{\infty} \phi^S_{v_\alpha}(x_\alpha - \Delta x_\alpha) \phi^T_{w_\alpha}(x_\alpha) dx_\alpha \right\}$$
$$+ \sum_{\alpha=2}^{6} b^i_\alpha \left[\int_{-\infty}^{\infty} \phi^S_{v_\alpha}(x_\alpha - \Delta x_\alpha) x_\alpha \phi^T_{w_\alpha}(x_\alpha) dx_\alpha \right] \left\{ \prod_{\beta \neq \alpha} \left[\int_{-\infty}^{\infty} \phi^S_{v_\beta}(x_\beta - \Delta x_\beta) \phi^T_{w_\beta}(x_\beta) dx_\beta \right] \right\}$$

where the *T* superscripts have been removed from the normal modes for clarity. The EOM-CCSD/6-311G formalism was used to evaluate the electronic contributions, where the SOC matrix element $\langle \psi_S | \hat{H}_{SO} | \psi_T \rangle_i$ and derivative couplings b^i_α were taken to be those obtained at the initial (singlet) state geometry. The derivative terms were obtained from the SOC curves given in Figure S18; b^S_α values were obtained by taking the derivatives at the appropriate values of Δx_α given in Table S8. The SOC calculations at the equilibrium geometry gives $\langle \psi_S | \hat{H}_{SO} | \psi_T \rangle_S / hc$= 33.86 cm^{-1}. We note that similar results are obtained when the triplet geometry is chosen as the reference point.

In practice, calculating these rates requires restricting our consideration to a limited range of vibrational levels, which we do here by only considering SP(S_1, $\vec{v}$) and SP(T_0, $\vec{w}$) states with $\sum v_\alpha, w_\alpha \leq v_{max}$. Table S9 shows the nonzero total rates, Eq. (S6), out of the singlet manifold for a choice of $v_{max} = 3$; even this limited set of possible initial and final states produces a considerable number of unexpectedly high ISC rates. Most notably, no vibrational excitation is required for transition from SP(S_1) to the triplet SP(T_0), with the v_{max} $= 3$ result giving a time constant $\tau_{S0}^{SV} = 1/k_{S0}^{SV}$ of ca. 0.91 ps. The total ISC rate out of the SP(S_1) vibrational ground state is provided in Table S10 for several choices of v_{max}, showing that this time constant is further decreased when a larger number of final states are included in the calculation, with the time constant dropping to ca. 0.5 ps when states with up to 10 quanta of excitation are considered.

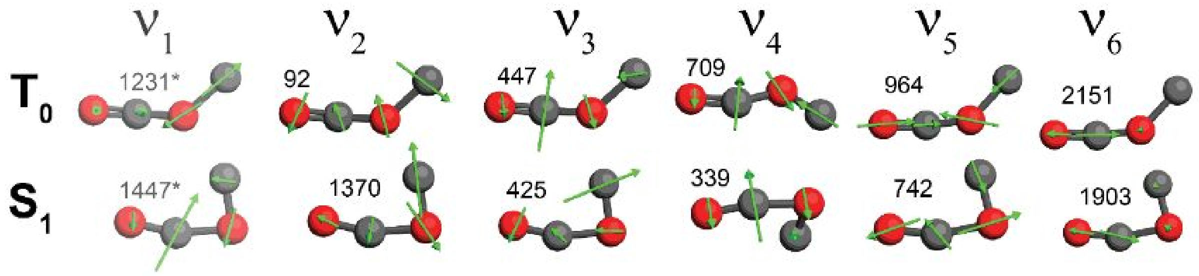

Figure S17. Parallel approximation for SP(S_1) and SP(T_0). Normal modes of SP(T_0) (top) and SP(S_1) (bottom) obtained from ωB97M-V/6-311+G* are illustrated, with frequencies in units of cm^{-1}. Imaginary-frequency modes are indicated with an asterisk.

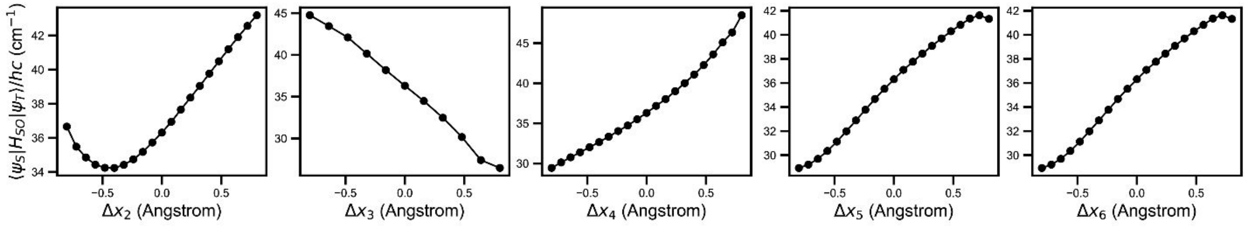

Figure S18. Nuclear dependence of SOC between SP states. EOM-CCSD/6-311G SOC elements $\langle\psi_S|\hat{H}_{SO}|\psi_T\rangle/hc$ between the triplet and singlet states of the saddle points are evaluated at geometries displaced from the SP(T_0) equilibrium along each of the five normal modes used to calculate the SV-ISC rates.

Table S8. Mode-specific parameters used to calculate SV-ISC rates between vibronic levels of SP(T_0) and SP(S_1). All values are obtained from the ωB97M-V and EOM-CCSD calculations. Derivative coupling elements b_α^S were determined by taking the derivatives of the curves in Fig. S18 at the indicated values of Δx_α.

α	b_α^S/hc cm^{-1} Å^{-1}	Δx_α Å	ω_α^T cm^{-1}	μ_α^T amu	ω_α^S cm^{-1}	μ_α^S amu
2	-12.65	-0.762	92.16	13.8492	1369.81	13.5300
3	-12.27	0.191	446.80	12.8714	424.85	12.9790
4	15.50	0.489	708.89	13.0915	339.03	13.0748
5	9.48	0.067	963.69	15.0778	741.50	14.8973
6	4.43	-0.053	2150.72	13.0403	1902.90	13.2366

Table S9. ISC rates out of SP(S_1). Total SV- and FC-ISC rates ($k_{S\vec{v}}^{SV}$ and $k_{S\vec{v}}^{FC}$, respectively) are given in units of s^{-1} for transitions originating in the indicated vibrational level $\vec{v}$ of SP(S_1) where $\sum v_\alpha, w_\alpha \leq 3$. The energies of each initial state relative to the ZPE of SP(S_1), as well as the ZPE of 2CO($\tilde{X}\ ^1\Sigma^+$) obtained from Table S1, are given in units of cm^{-1} and eV, respectively.

v_2	v_3	v_4	v_5	v_6	$E_{\vec{v}}^S - E_{\vec{0}}^S$	$E_{\vec{v}}^S -$ 2CO	$k_{S\vec{v}}^{FC}$	$k_{S\vec{v}}^{SV}$	v_2	v_3	v_4	v_5	v_6	$E_{\vec{v}}^S - E_{\vec{0}}^S$	$E_{\vec{v}}^S -$ 2CO	$k_{S\vec{v}}^{FC}$	$k_{S\vec{v}}^{SV}$
0	0	0	0	3	5709	7.47	2.1E12	3.6E12	0	0	1	0	0	339	6.80	1.4E10	6.8E9
0	2	0	0	0	850	6.86	5.6E11	1.5E12	0	1	1	0	1	2667	7.09	1.6E9	5.7E9
0	0	0	0	0	0	6.76	4.1E11	1.1E12	1	0	1	1	0	2450	7.06	2.2E9	5.4E9
0	1	0	0	0	425	6.81	2.4E11	6.5E11	2	0	0	0	0	2740	7.10	2.1E9	4.2E9
0	1	0	1	1	3069	7.14	5.4E10	2.3E11	1	0	0	1	0	2111	7.02	3.7E9	3.1E9
0	1	1	0	0	764	6.85	6.8E10	2.1E11	1	1	0	0	0	1795	6.98	1.9E9	1.1E9
0	0	1	2	0	1822	6.98	1.6E11	2.0E11	0	0	0	1	1	2644	7.09	9.9E8	7.5E8
0	1	0	0	2	4231	7.28	3.9E10	1.7E11	1	0	0	0	1	3273	7.16	8.5E8	7.0E8
0	0	0	1	0	742	6.85	1.5E11	1.3E11	1	0	2	0	0	2048	7.01	1.8E9	6.8E8
0	0	1	1	1	2983	7.13	9.7E10	1.3E11	1	2	0	0	0	2220	7.03	1.0E9	4.8E8
0	0	0	0	1	1903	6.99	1.0E11	8.4E10	0	1	0	2	0	1908	6.99	2.2E8	4.2E8
0	0	1	0	2	4144	7.27	5.0E10	6.6E10	0	2	1	0	0	1189	6.91	4.8E7	3.0E8
0	2	0	1	0	1591	6.96	7.5E9	4.1E10	2	0	1	0	0	3079	7.14	9.8E7	2.0E8
0	0	3	0	0	1017	6.88	3.9E10	3.8E10	0	3	0	0	0	1275	6.92	1.7E7	1.9E8
0	0	1	1	0	1081	6.89	1.7E10	3.5E10	0	0	0	3	0	2225	7.03	5.6E7	1.0E8
0	1	0	1	0	1166	6.90	5.8E9	3.2E10	1	1	1	0	0	2134	7.02	2.9E7	8.9E7
1	0	0	0	0	1370	6.93	2.0E10	2.7E10	2	0	0	1	0	3481	7.19	3.0E7	1.9E7
0	0	2	0	0	678	6.84	1.4E10	2.6E10	0	0	0	2	1	3386	7.18	5.2E6	9.4E6
0	1	1	1	0	1505	6.94	7.2E9	2.6E10	1	1	0	1	0	2536	7.07	2.3E7	7.9E6
0	2	0	0	1	2753	7.10	3.1E9	1.7E10	3	0	0	0	0	4109	7.27	5.8E6	5.4E6
0	1	2	0	0	1103	6.89	7.7E9	1.4E10	0	0	2	1	0	1420	6.93	3.6E7	3.7E6
0	1	0	0	1	2328	7.05	2.3E9	1.2E10	2	1	0	0	0	3164	7.15	2.4E5	6.6E5
0	0	1	0	1	2242	7.04	6.8E9	9.4E9	1	0	0	1	1	4014	7.26	1.6E7	5.0E5
0	0	0	2	0	1483	6.94	8.7E9	7.7E9	1	0	1	0	0	1709	6.97	1.3E7	1.3E5
1	0	1	0	1	3612	7.21	3.1E9	7.7E9									

Table S10. ISC rates out of the vibrational ground state of SP(S_1). Total SV- and FC-ISC rates ($k_{S\vec{0}}^{SV}$ and $k_{S\vec{0}}^{FC}$, respectively) as well as the corresponding time constants (given by the inverse of the corresponding rate) for transitions SP(S_1,$\vec{0}$)⇝SP(T_0) where we restrict our calculations to include all final states SP(T_0, $\vec{w}$) with $\sum w_\alpha \leq v_{max}$. The total number of final states is also given, as is the energy (in cm^{-1}) of the highest level relative to SP(T_0, $\vec{0}$). Further increasing v_{max} was not found to change the resultant rate (for up to v_{max} = 15).

v_{max}	total states	max. E	$k_{S\vec{0}}^{FC}$ (s^{-1})	$\tau_{S\vec{0}}^{FC}$ (ps)	$k_{S\vec{0}}^{SV}$ (s^{-1})	$\tau_{S\vec{0}}^{SV}$ (ps)
3	56	6452	4.1E11	2.5	1.1E12	0.91
5	252	10754	4.2E11	2.4	1.1E12	0.90
10	3003	21507	8.4E11	1.2	1.9E12	0.53

b. Other CO Dimer Structures

While the above treatment shows that rapid ISC is possible between vibrational levels of the saddle points identified for reaction **[1]**, there are several other points on the singlet surface which may be considered to provide access to the triplet pathway. In particular, the MECP identified in Section S5 seems an intuitive choice. However, a frequency analysis at the ωB97M-V/6-311+G* level of theory yields two imaginary frequencies for this geometry in the singlet state (Table S3); thus, application of the ISC frameworks considered here would require further limiting the dimensionality of the nuclear wavefunctions. This would most likely lead to an over-estimation of the Franck-Condon factors describing the nuclear contributions to the overall rates. Given the strong resemblance between SP(S_1) and the singlet MECP, we consider the rates calculated above to be a reasonable estimate of ISC transitions out of dimer structures resembling the singlet MECP, and note that the DFT treatment of the gas-phase model should serve only as a qualitative guide to the processes occurring in the crystal samples studied here.

The identification of the cyclic IM(S_1) provides an additional possible means by which the triplet surface may be reached, via relaxation to the linear IM(T_0) OCCO geometry. However, as noted by Jamieson and coworkers,[7] this structure has not been observed experimentally, and our work indicates that if IM(S_1) is formed it should rapidly reconvert to the 2CO reactants (Fig. S16, Section S4b). Additionally, the large difference in geometries for the singlet/triplet structures indicates that the Franck-Condon overlap would be poor, leading to low ISC rates. Thus, we do not consider IM(S_1) as a likely point of access for the product-forming triplet pathway.

REFERENCES

1. Lee, T. J.; Taylor, P. R. A diagnostic for determining the quality of single-reference electron correlation methods. *Int. J. Quantum Chem.* **1989,** *36*, 199-207.
2. Berg, O.; Ewing, G. E. The determination of monolayer structure by infrared-spectroscopy - CO_2 on NaCl(100). *Surf. Sci.* **1989,** *220*, 207-229.
3. Gerakines, P. A.; Schutte, W. A.; Ehrenfreund, P. Ultraviolet processing of interstellar ice analogs. 1. Pure ices. *Astron. Astrophys.* **1996,** *312*, 289-305.
4. Gerakines, P. A.; Moore, M. H. Carbon suboxide in astrophysical ice analogs. *Icarus* **2001,** *154*, 372-380.
5. Trottier, A.; Brooks, R. L. Carbon-chain oxides in proton-irradiated CO ice films. *Astrophys. J.* **2004,** *612*, 1214-1221.

6. Loeffler, M. J.; Baratta, G. A.; Palumbo, M. E.; Strazzulla, G.; Baragiola, R. A. CO_2 synthesis in solid CO by Lyman-α photons and 200 keV protons. *Astron. Astrophys.* **2005,** *435*, 587-594.
7. Jamieson, C. S.; Mebel, A. M.; Kaiser, R. I. Understanding the kinetics and dynamics of radiation-induced reaction pathways in carbon monoxide ice at 10 K. *Astrophys. J. Suppl. S.* **2006,** *163*, 184-206.
8. Ciaravella, A.; Chen, Y. J.; Cecchi-Pestellini, C.; Jimenez-Escobar, A.; Caro, G. M. M.; Chuang, K. J.; Huang, C. H. Chemical evolution of a CO ice induced by soft x-rays. *Astrophys. J.* **2016,** *819*, 38.
9. Chang, H. C.; Richardson, H. H.; Ewing, G. E. Epitaxial-growth of CO on NaCl(100) studied by infrared-spectroscopy. *J. Chem. Phys.* **1988,** *89*, 7561-7568.
10. Jiang, G. J.; Person, W. B.; Brown, K. G. Absolute infrared intensities and band shapes in pure solid CO and CO in some solid matrices. *J. Chem. Phys.* **1975,** *62*, 1201-1211.
11. Kim, K. H.; Lee, B.; Lee, S. Structures and spectroscopic properties of OC_nO (n=2-6): Density functional theory study. *B. Kor. Chem. Soc.* **1998,** *19*, 553-557.
12. Hudgins, D. M.; Sandford, S. A.; Allamandola, L. J.; Tielens, A. G. G. M. Midinfrared and far-infrared spectroscopy of ices - Optical-constants and integrated absorbances. *Astr. Soc. P.* **1993,** *41*, 271-272.
13. Chen, L.; Lau, J. A.; Schwarzer, D.; Meyer, J.; Verma, V. B.; Wodtke, A. M. The Sommerfeld ground-wave limit for a molecule adsorbed at a surface. *Science* **2019,** *363*, 158-161.
14. Krupskii, I. N.; Prokhvatilov, A. I.; Erenburg, A. I.; Yantsevich, L. D. Structure and thermal expansion of α-CO. *Phys. Stat. Sol. (a)* **1973,** *19*, 519-527.
15. Lau, J. A.; Chen, L.; Choudhury, A.; Schwarzer, D.; Verma, V. B.; Wodtke, A. M. Transporting and concentrating vibrational energy to promote isomerization. *Nature* **2021,** *589*, 391-395.
16. Barreto, P. R. P.; Euclides, H. D.; Albernaz, A. F.; Aquilanti, V.; Capitelli, M.; Grossi, G.; Lombardi, A.; Macheret, S.; Palazzetti, F. Gas phase Boudouard reactions involving singlet-singlet and singlet-triplet CO vibrationally excited states: Implications for the non-equilibrium vibrational kinetics of CO/CO_2 plasmas. *Eur. Phys. J. D* **2017,** *71*, 259.
17. Shao, Y. H.; Head-Gordon, M.; Krylov, A. I. The spin-flip approach within time-dependent density functional theory: Theory and applications to diradicals. *J. Chem. Phys.* **2003,** *118*, 4807-4818.
18. Maeda, S.; Ohno, K.; Morokuma, K. Updated branching plane for finding conical intersections without coupling derivative vectors. *Journal of Chemical Theory and Computation* **2010,** *6*, 1538-1545.
19. Hall, B. O.; James, H. M. Lattice dynamics of α carbon monoxide. *Phys. Rev. B* **1976,** *13*, 3590-3603.
20. Huber, K. P.; Herzberg, G. H., Constants of Diatomic Molecules. In *NIST Chemistry WebBook, NIST Standard Reference Database Number 69*, National Institutes of Standards and Technology: Gaithersburg MD, 2021.
21. Marian, C. M. Understanding and controlling intersystem crossing in molecules. *Annu. Rev. Phys. Chem.* **2021,** *72*, 617-640.
22. Penfold, T. J.; Gindensperger, E.; Daniel, C.; Marian, C. M. Spin-vibronic mechanism for intersystem crossing. *Chem. Rev.* **2018,** *118*, 6975-7025.
23. Serdyukov, A.; Vetter, M.; Brodyanski, A.; Jodl, H. J. Lattice phonons of solid phases (α, β, δ, ε) of carbon monoxide by optical studies. *Low Temp. Phys.* **2010,** *36*, 424-438.
24. Duschinsky, F. The importance of the electron spectrum in multi atomic molecules. concerning the Franck-Condon principle. *Acta Physicochimica U.R.S.S.* **1937,** *7*, 551-566.
